# Online, Group-Based Psychological Support for Adolescent and Young Adult Cancer Survivors: Results from the Recapture Life Randomized Trial

**DOI:** 10.3390/cancers13102460

**Published:** 2021-05-18

**Authors:** Ursula M. Sansom-Daly, Claire E. Wakefield, Sarah J. Ellis, Brittany C. McGill, Mark W. Donoghoe, Phyllis Butow, Richard A. Bryant, Susan M. Sawyer, Pandora Patterson, Antoinette Anazodo, Megan Plaster, Kate Thompson, Lucy Holland, Michael Osborn, Fiona Maguire, Catherine O’Dwyer, Richard De Abreu Lourenco, Richard J. Cohn, The Recapture Life Working Party

**Affiliations:** 1 School of Women’s and Children’s Health, UNSW Sydney, Kensington, NSW 2033, Australia; c.wakefield@unsw.edu.au (C.E.W.); sarah.ellis@unsw.edu.au (S.J.E.); b.mcgill@unsw.edu.au (B.C.M.); m.donoghoe@unsw.edu.au (M.W.D.); antoinette.anazodo@unsw.edu.au (A.A.); r.cohn@unsw.edu.au (R.J.C.); 2 Behavioural Sciences Unit, Kids Cancer Centre, Sydney Children’s Hospital, Randwick, NSW 2031, Australia; 3 Sydney Youth Cancer Service, Nelune Comprehensive Cancer Centre, Prince of Wales Hospital, Randwick, NSW 2031, Australia; Fiona.Maguire@health.nsw.gov.au (F.M.); Cath.ODwyer@health.nsw.gov.au (C.O.); 4 Stats Central, Mark Wainwright Analytical Centre, UNSW Sydney, Kensington, NSW 2033, Australia; 5 Centre for Medical Psychology & Evidence-Based Decision-Making (CeMPED), School of Psychology, University of Sydney, Sydney, NSW 2050, Australia; phyllis.butow@sydney.edu.au; 6 School of Psychology, UNSW Sydney, Kensington, NSW 2033, Australia; r.bryant@unsw.edu.au; 7 Department of Paediatrics, The University of Melbourne, Melbourne, VIC 3052, Australia; susan.sawyer@rch.org.au; 8 Royal Children’s Hospital Centre for Adolescent Health, Melbourne, VIC 3052, Australia; 9 Murdoch Children’s Research Institute, Melbourne, VIC 3052, Australia; 10 Research, Evaluation and Policy Unit, CanTeen, Sydney, NSW 2042, Australia; pandora.patterson@canteen.org.au; 11 Faculty of Medicine and Health, The University of Sydney, Sydney, NSW 2050, Australia; 12 Kids Cancer Centre, Sydney Children’s Hospital, Randwick, NSW 2031, Australia; 13 Western Australia Youth Cancer Service, Sir Charles Gairdner Hospital, WA 6009, Australia; Megan.Plaster@health.wa.gov.au; 14 Victorian Adolescent & Young Adult Cancer Service, Peter MacCallum Cancer Centre, Melbourne, VIC 3000, Australia; kate.thompson@petermac.org; 15 Department of Social Work, The University of Melbourne, Melbourne, VIC 3010, Australia; 16 Queensland Child and Youth Clinical Network, Clinical Excellence Queensland, Herston, QLD 4006, Australia; Lucy.Holland@qut.edu.au; 17 School of Nursing, Queensland University of Technology, Brisbane, QLD 4000, Australia; 18 Youth Cancer Service SA/NT, Royal Adelaide Hospital, Adelaide, SA 5000, Australia; michael.osborn@sa.gov.au; 19 Centre for Health Economics Research and Evaluation, University of Technology Sydney, Haymarket, NSW 2000, Australia; richard.deabreulourenco@chere.uts.edu.au

**Keywords:** adolescent, young adult, survivor, cancer survivorship, psychological interventions, online videoconferencing, telehealth, cognitive-behavioral therapy, quality of life, cancer continuum

## Abstract

**Simple Summary:**

Adolescents and young adult cancer survivors are vulnerable to psychological distress after completing cancer treatment. Telehealth (online videoconferencing) interventions may be able to address the gap in tailored, evidence-based supportive interventions. We evaluated an online, group-based, videoconference-delivered cognitive-behavioral therapy intervention (‘Recapture Life’) in a randomized trial. Forty cancer survivors between the ages of 15–25 years participated. No positive impacts on participants’ quality of life emerged immediately following the intervention, but Recapture Life participants reported using more adaptive coping skills. Recapture Life participants also reported higher negative impact of cancer, anxiety and depression at a 12-month follow-up. Additional analyses suggested that survivors benefitted differently from the two online interventions (Recapture Life vs. peer-support group) depending on how recently they had completed their cancer treatment. Our data highlight that different survivor sub-groups may find group-based, telehealth psychological interventions more or less helpful at different points in survivorship.

**Abstract:**

Telehealth interventions offer a practical platform to support adolescent and young adult (AYA) cancer survivors’ mental health needs after treatment, yet efficacy data are lacking. We evaluated an online, group-based, videoconferencing-delivered cognitive-behavioral therapy (CBT) intervention (‘Recapture Life’) in a 3-arm randomized-controlled trial comparing Recapture Life with an online peer-support group, and a waitlist control, with the aim of testing its impact on quality of life, emotional distress and healthcare service use. Forty AYAs (M_age_ = 20.6 years) within 24-months of completing treatment participated, together with 18 support persons. No groupwise impacts were measured immediately after the six-week intervention. However, Recapture Life participants reported using more CBT skills at the six-week follow-up (OR = 5.58, 95% CI = 2.00–15.56, *p* = 0.001) than peer-support controls. Recapture Life participants reported higher perceived negative impact of cancer, anxiety and depression at 12-month follow-up, compared to peer-support controls. Post-hoc analyses suggested that AYAs who were further from completing cancer treatment responded better to Recapture Life than those who had completed treatment more recently. While online telehealth interventions hold promise, recruitment to this trial was challenging. As the psychological challenges of cancer survivorship are likely to evolve with time, different support models may prove more or less helpful for different sub-groups of AYA survivors at different times.

## 1. Introduction

Adolescent and young adult (AYA) cancer survivors experience high rates of common mental health difficulties, such as depression and anxiety disorders [1,2]. For these survivors, the documented stresses associated with their cancer diagnosis collide with an already-challenging developmental period [1,3]. The risk of onset of mental health concerns peaks during the AYA years, with 75% of mental disorders commencing by the age of 24 years [4,5]. Developing and evaluating interventions to best meet AYA cancer survivors’ mental health needs is recognized as an international clinical and research priority [6,7,8]. This sits within a broader policy context promoting preventative approaches to youth mental health [9,10].

Considerable advances have been made in the development and implementation of age-appropriate, AYA-specific clinical and hospital-based services over the past decade [6,11,12,13,14,15]. However, these services typically focus on AYAs in active phases of cancer treatment. Meeting AYAs’ post-cancer care mental health needs can be more difficult as young people leave hospital-based environments to return to their home communities, which may be geographically distant from their treating center [16]. Delivering mental health interventions in AYAs’ local communities may best support AYAs’ re-integration back into life after cancer [17]. In addition to the practical benefits from reduced travel and its interruption to daily routines, community-based delivery of post-treatment support may also reduce AYAs’ identity as a sick ‘patient’ and facilitate the transition towards a post-cancer identity as a capable, well-functioning adult [1,18].

Online telehealth interventions offering community-based mental health care using videoconferencing technology have proliferated in recent years, and appear ideally placed to meet the unique needs of AYA cancer survivors [19,20,21]. Concerns around the recent global coronavirus (COVID-19) pandemic have further reinforced the infectious control benefits of remotely-delivered, telehealth interventions, including for young people living with and beyond cancer [22,23]. Telehealth interventions can enhance equity in accessing support by obviating the need for travel (which can be difficult for those with physical symptoms such as pain, fatigue and immobility), overcome the stigma around help-seeking for mental health (which is particularly pertinent for AYAs) by providing a private and individually-tailored mode of delivery [22,24,25,26,27], and broaden the geographic reach of specialist psycho-oncology services that are often clustered in major cities. Recent data indicate that >85% of households in Australia [28], the US [29], and UK [30] have access to the internet, which suggests that dissemination of telehealth models would also be feasible at scale [21].

Clinical and research communities have welcomed online interventions, including in oncology [31,32]. Early evidence supports their acceptability and utility, both to cancer survivors and professionals [26,33,34,35]. Safety and feasibility data are however, only just emerging in AYA cancer survivors [35,36], while efficacy data are lacking. There is strong evidence that cognitive-behavioral therapy (CBT) is effective in addressing mental health disorders, such as anxiety and depression among AYAs outside of oncology, including among young people with sub-clinical distress [37,38,39]. However, this therapeutic approach has yet to be fully evaluated in the AYA oncology context [19,40]. Finally, while it is thought that online mental health interventions may reduce the costs incurred both by their users and those delivering them, little cost data are currently available [41,42]. Whether and how online interventions may impact AYAs’ subsequent need or desire to use other health- and mental healthcare services is also unknown.

Furthermore, it is likely that some individuals may respond better to online telehealth interventions than others [22], but the question of ‘who’ responds best remains unknown. The optimal type and timing of online interventions for AYA cancer survivors are also unclear: reviews report mixed results across different types of online support (including websites, videoconferencing-based telehealth interventions, and mobile-phone apps) with efficacy data often lacking [19,31,40]. AYAs can also experience heightened distress at several points from cancer diagnosis through to long-term survivorship [1].

We aimed to fill these evidence gaps through a rigorous evaluation of the Recapture Life intervention [43]. Recapture Life is a group-based, videoconferencing-delivered psychological intervention for AYA cancer survivors, facilitated by a psychologist. Informed by adolescent resilience models [44], it uses evidence-based CBT coping strategies, tailored to the key concerns of AYAs [36,43]. Recapture Life is delivered at treatment completion, recognized as a challenging period in the cancer trajectory, and a time when distress can peak and mental health concerns emerge [1,16]. This approach (deliberately targeting groups known to be vulnerable to developing mental health disorders by virtue of particular risk factors, in this case, a cancer diagnosis) is often referred to as ‘selective-preventative’ [45,46].

We have previously reported that Recapture Life is feasible and safe to deliver, as well as acceptable to AYAs and psychologists [33,36]. Here, we examine the efficacy and cost of Recapture Life in the early cancer survivorship period, compared to both an active peer-support group control and a waitlist control, within a three-arm randomized-controlled trial (RCT). Peer-support groups are commonly used in oncology, and may be particularly beneficial for AYAs from a developmental perspective. However, they have not yet been rigorously evaluated [19,47]. The use of the peer-support control group was designed to disentangle any specific benefits of group-based CBT, from the more general, non-specific supportive benefits of group-based peer-support. This gold-standard trial design also controlled for participants’ expectations of treatment effects [48]. We included a six-week waitlist arm to control for the possibility that AYAs’ distress may change in the early post-treatment period.

Compared to an active (peer-support group) and a waitlist control, the primary outcome of this study was to measure the impact of Recapture Life on quality of life at six-weeks (end-of-treatment) and 12-months later. Given the preventative, resilience-focused approach taken, the primary outcome of quality of life was designed to capture the potential for this support model to ameliorate the impact of cancer on a range of important facets of AYAs’ psychosocial adjustment into early survivorship. Secondary outcome measures were:

1. Psychological outcomes: Depression and anxiety symptoms;
2. Psychological processes/mechanisms: Coping skills, family functioning, cancer-related identity;
3. Intervention delivery: Fidelity, group cohesion, and working (therapeutic) alliance;
4. Health economics: AYA real-world functioning (including engagement in productive activities such as work and study), health service use, and medication use, costs of delivering Recapture Life, and any averted travel costs for AYA participants.

This paper presents the Recapture Life RCT data addressing the primary and all secondary outcomes up to 12 months post-intervention.

## 2. Materials and Methods

### 2.1. Study Design

**Figure 1:**
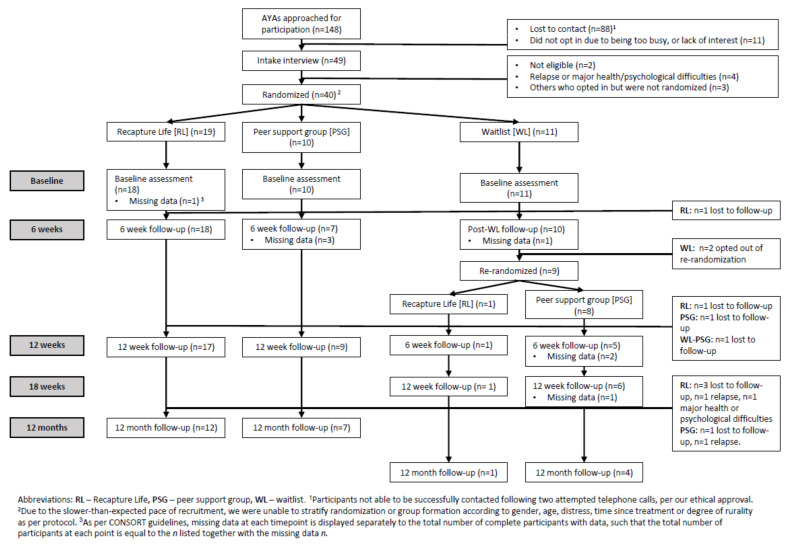
CONSORT flowchart depicting recruitment across Recapture Life trial.

Figure 1 depicts our three-arm, phase II feasibility RCT design (Australian and New Zealand Clinical Trials Registry reference: ACTRN12610000717055). Our study had ethical approval from the South Eastern Sydney Local Health District (Reference HREC/12/POWH/136).

#### 2.1.1. Randomization and Blinding

Randomization involved two separate algorithms, both generated by staff at the School of Psychology, UNSW (who were independent from the hospital-based research team). An algorithm was used to generate a random sequence (n = 15) of the three trial arms (Recapture Life, peer-support group, and waitlist), equally divided across the three arms. Groups of five consecutively recruited participants were allocated to each randomly-generated condition, before moving to the next with the subsequent group of five participants. This approach minimizes the waiting time for individual participants before an online group matching their allocation can be run with sufficient participant numbers, reducing drop-out and participant burden [49]. Waitlisted participants, at the conclusion of their waitlist, were re-randomized to receive either Recapture Life or the peer-support group, using a similar randomization algorithm at the group level. Re-randomized participants could be allocated to join online groups formed together with participants being randomized for the first time. Participants were blinded to their treatment allocation, however trial psychologists and research assistants could not be blinded due to the distinctness of intervention materials. Two psychologists facilitated groups across both arms (Recapture Life and peer-support group) to prevent any confounding of intervention- and psychologist-specific delivery factors.

#### 2.1.2. Sample Size and Power Calculation

We calculated that a total sample size of 90 (assuming equal numbers in each group) would allow a medium-large effect size [50] of *d* = 0.65 (calculated as the difference in change from T1 to T2 for any pair of groups, standardized on the pooled within-group standard deviation, and assuming a correlation between T1 and T2 of 0.6) on the primary outcome variable (QoL) to be detected with a power of 80% at a two-tailed significance level of 0.05. This clinically-significant [51] effect size was based on RCTs of similar interventions for AYAs with chronic illness [52,53,54] and online interventions targeted toward this age group [55]. Assuming a response rate of 50% and attrition rate of 20%, which appeared feasible based on pilot work by our team, we originally anticipated that we would need to approach approximately 220 patients to achieve a final sample of 90 participants [56].

#### 2.1.3. Participants and Recruitment

In line with Australian Youth Cancer Service definitions, eligible AYAs were defined as aged 15–25 years at the time of (curative) treatment completion and who finished treatment no more than 24 months ago. We recruited these participants through four pediatric and seven adult hospitals across Australia, as well as through three community organizations, via an invitation letter from their oncologist/local health-care professional. Collaborating site-specific clinicians were responsible for identifying eligible AYAs to invite from their site. AYAs were ineligible if they (i) did not speak adequate English (determined by either the need for clinical interpreters; or through AYA self-report during the intake process); (ii) demonstrated severe distress (e.g., active suicidality, psychosis, and/or extremely severe depression), or (iii) had an incurable cancer diagnosis.

Participating AYAs were also given the opportunity to invite any support person (e.g., parent, partner/spouse) over the age of 18 years to receive psycho-education about the intervention, to help them better support the young person and their engagement with the intervention. Support persons also completed questionnaires as part of the study

### 2.2. Trial Arms

#### 2.2.1. The Recapture Life Intervention

The development of Recapture Life has been previously described in detail [43]. In brief, the online program involved six once-weekly 90-min small-group sessions involving 3–5 AYAs per group, facilitated by a psychologist, using online videoconferencing software (WebEx, by Cisco; San Jose, CA, USA). Each week, the group explored common cancer survivorship experiences, and relevant evidence-based CBT coping strategies. Participants were provided with a Recapture Life workbook containing psycho-educational content reflective of the online sessions, and home-practice activities to support mastery of the coping strategies discussed.

Participating support persons received a single telephone-based consultation with the psychologist facilitating their AYA’s group, and weekly email updates of intervention content. As well as describing key session content, these updates provided generic examples of ways support persons could support the AYA and communicate about the weekly topic. Table 1 summarizes the content of Recapture Life and peer-support group programs.

#### 2.2.2. The Non-Directive, Peer-Support Group Control (Active Control)

The peer-support group control matched Recapture Life in terms of frequency, type of contact and inclusion of support persons. However, instead of actively teaching and discussing CBT-related coping strategies, the peer-support group sessions focused on a different cancer-survivorship ‘theme’ each week, and involved an exclusive focus on non-directive, supportive group discussion between the AYA survivors [43].

#### 2.2.3. Waitlist

AYAs randomized to waitlist completed the first baseline assessment, were waitlisted for six weeks, completed a repeat baseline assessment, and were then re-randomized to receive either Recapture Life or peer-support group at the conclusion of their waitlist period.

### 2.3. Measures

Self-reported measures were used to assess the impact of Recapture Life for both AYAs and support person participants, using online or paper-based questionnaires (according to participant preference), with additional detail previously published [43]. Table 2 details the administration schedule, scoring and psychometric properties for these measures in this population.

#### 2.3.1. Primary Outcome

Quality of life. Five subscales from the *Impact of Cancer Scale* AYA module [58,59], assessed positive and negative impacts of cancer (Table 2). For all outcomes, higher subscale scores indicate a greater impact of cancer.

#### 2.3.2. Secondary Outcomes

##### Psychological Outcomes

Depression and anxiety symptoms. We included the Depression, Anxiety and Stress Scale-Short Form [60] depression and anxiety subscales.

##### Psychological Mechanisms

Identity changes. We used the Centrality of Events Scale-Short Form [61], where a higher total score indicates that a stressful/traumatic life event is more central to a person’s identity and life story. Participants also rated the extent to which they viewed themselves as either a cancer “patient” or “survivor” using a study-developed 10-point survivor identity visual analogue scale.

Unmet cancer-related needs. We used a subset of 17 items from the Cancer Needs Questionnaire for Parents/Carers of Adolescents and Young Adults with Cancer [62] to gauge support person unmet needs over time.

##### Coping Strategies

Positive and negative coping approaches. The KIDCOPE (Older version) [63] measured positive and negative adolescent coping approaches. A second study-developed scale assessed participants’ CBT skills acquisition and use regarding 10 specific skills important in survivorship.

Family functioning. Both AYAs and support person participants completed three subscales of the *McMaster Family Assessment Device* [64].

##### Intervention Delivery Factors

Manual fidelity. Psychologist facilitators self-reported their fidelity to the Recapture Life manual following each session, and two blinded independent assessors also reviewed session recordings (Supplementary Data S1).

Working alliance. We used four items of the Working Alliance Inventory-Short Form [65].

Group cohesion. This was measured by four items from the validated California Psychotherapy Alliance Scale for Group [66].

Benefit and burden of program. Two validated items [67] assessed perceived benefit and burden of participating in the study. Additional free-text comments were optional.

##### Health Economics Outcomes: Cost and Real-World Functioning (Study-Developed Items)

Real-world functioning. Two items asked AYAs/support persons’ general functioning through engagement in, and absenteeism from, real-world activities.

Health, mental health service, and medication use. Participants reported whether they had used a range of health services over the past six months. Participants also reported whether they were currently taking any medications/supplements, and to indicate the reason for their use.

Cost. (i) Cost of delivery. We calculated the cost of delivering Recapture Life by analyzing a detailed log of all participant clinical contacts (both number and duration) relevant to the appropriate clinical delivery of the program during the trial. The costs used reflected the level of expertise appropriate for the delivery of the intervention, across a range of clinical-academic settings (Supplementary Data S2).

(ii) Estimated travel costs saved for AYAs. We also estimated travel costs averted for our Recapture Life AYA participants by calculating the estimated additional costs that would have been borne by participants if they had to travel to their local hospital site to attend a similar number of intervention sessions as in Recapture Life (Supplementary Data S2).

### 2.4. Data Analysis

We used R version 3.6.0 [88] to perform all analyses. Descriptive statistics were used to summarize participant characteristics. Abiding by the intention-to-treat principle [89], we performed comparisons between trial arms in two ways: Changes between baseline and the 6-week follow-up were compared across all three groups (three-way analyses: examining outcomes across Recapture Life, peer-support group control, and waitlist control), while changes over the 12-month follow-up period were compared between the Recapture Life and peer-support arms, with waitlisted participants analyzed according to the active treatment arm on which they were re-randomized, and their outcomes at the post-waitlist follow-up being used as their baseline (two-way analyses).

For comparisons of continuous outcomes (impact of cancer, depression, anxiety, centrality of events and family functioning), we used the nlme package [90] to fit linear mixed effects models with individual-specific intercepts and a continuous AR(1) residual structure, and employed likelihood ratio tests to assess the differences between arms in average changes over time, accounting for baseline. For other outcomes, we used the lme4 [91] and ordinal [92] packages to fit generalized linear mixed effects models similar to the linear mixed model previously described. We used binomial logistic (coping strategies, CBT skills, unmet cancer needs, health service use), ordinal logistic (benefit, burden, survivor identity), or log-link Poisson (number of emergency department visits, nights in hospital) models, as appropriate. Fitted values and their 95% confidence intervals from each model were produced using the emmeans package [93]. These post-hoc exploratory analyses are typically underpowered, and non-significant results in these analyses does not necessarily mean there is no effect.

In order to better understanding whether particular subgroups of AYAs responded differently to the two online interventions, we also undertook several post-hoc exploratory moderation analyses to examine whether three key participant factors (age, sex, and time since treatment-completion) were associated with differences between arms in the primary outcome of interest (Impact of Cancer), as well as depression and anxiety symptoms. We did this by extending our mixed-effects models and performing likelihood ratio tests on three-way interactions between trial arm, time point and each characteristic of interest.

In cases where the outcome variable did not have a simple parametric distribution (days off work/study, days engaged with productive activities), we performed non-parametric Kruskal-Wallis rank sum tests on the changes from baseline at each time point. We used Chi-squared tests for simple comparisons of post-baseline relapses and manual fidelity between arms.

## 3. Results

### 3.1. Participants

Our final sample included 40 AYAs (27% of the 148 AYAs invited; 89% of 45 AYAs who opted-in), and 18 support persons (Figure 1) [36]. Our sample was ultimately recruited from April 2012-August 2015 from 10 sites; four hospitals (2 adult, 2 pediatric) did not recruit any participants [36,94]. The trial was stopped due to the project’s funding ending. Using the assumptions of our original power calculations, this final sample gave us 30% power to detect differences between waitlist and peer-support control arms, and 43% between waitlist and Recapture Life arms.

As previously published [36], the AYAs had a mean age of 20.6 years (SD = 3.0, median = 20 years, range: 15–26), had finished cancer treatment an average of 8 months previously (SD = 4.7 months; median = 7, range: 1–19) and were balanced by gender (female: *n* = 21, 52%). Half of the sample had been diagnosed with a hematological malignancy (*n* = 20, 50%). We have previously reported clinical challenges experienced during the trial [34], and while no harmful or adverse events occurred as a result of the trial or intervention, at study close, four participants (11.8%) reported a confirmed relapse (from intake to 12-month follow-up). Participants lived on average 82 km from their nearest capital city (SD = 124 km; median = 17; range = 3.8–429). Two-thirds of support persons were mothers (*n* = 12, 67%). Support persons had a mean age of 43.9 years (SD = 11.9, median = 49, range: 21–59). Table 3 depicts participant characteristics by RCT arm. Our 11 online groups mostly comprised a mix of AYA ages and genders (see Supplementary Data S3 for detail on group composition).

Most of our AYA sample (34, 91.9%) reported never having consulted mental health professionals prior to their cancer diagnosis (including psychologists, social workers, counsellors, or psychiatrists). However, over one-third (*n* = 14, 38.0%) reported having consulted mental health professionals at any time since their diagnosis, and prior to the study’s baseline

### 3.2. Primary Outcome: Impact of Cancer on Quality of Life

As noted above, we undertook three-way analyses (Recapture Life vs. peer-support group control vs. waitlist control at 6 weeks post-intervention) and two-way analyses (Recapture Life vs. peer-support group control 12-month post-intervention).

#### 3.2.1. Positive and Negative Impact of Cancer

No evidence of differences emerged between Recapture Life, peer-support group control, or waitlist control on AYAs’ perceptions of either the positive (all *p*-values ≥ 0.46) or negative (*p*-values ≥ 0.14) impact of cancer over the 6-week intervention period (see Supplementary Data S4)

**Figure 2:**
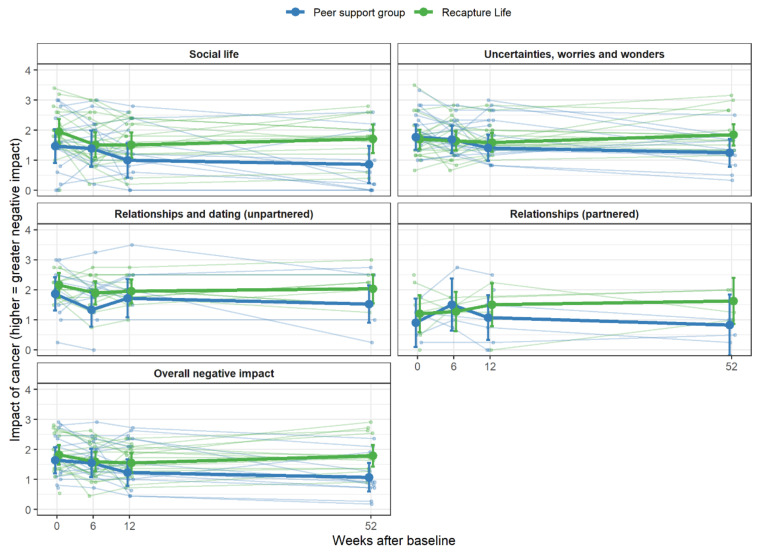
Differences between Recapture Life and peer-support group on negative impact of cancer, over time. Note. Individual scores are displayed, overlaid with thicker lines showing group-specific means and 95% confidence intervals. Three-way analyses comparing Recapture Life, peer-support group and waitlist are depicted in Supplementary Data S4).

However, Recapture Life participants reported higher perceptions of experiencing an overall negative impact of cancer (difference = 0.53, 95% CI = (0.03–1.03), *p* = 0.038) and impact on the cancer-related uncertainties subscale than peer-support group participants at 12 months post-intervention (difference = 0.67, 95% CI = (0.17–1.17), *p* = 0.009; Figure 2).

#### 3.2.2. Post-Hoc Moderation Analyses

Neither age nor time since cancer treatment-completion appeared to impact AYAs’ impact of cancer scores. However, post-hoc analyses provided some evidence suggesting that the positive impact of cancer differed with age, though this did not reach significance (*F_(1,48)_* = 2.88, *p* = 0.096); younger AYAs allocated to Recapture Life reported higher positive impact of cancer compared to peer support group control, with little difference between treatment arms for older AYAs (Supplementary Data S5).

### 3.3. Secondary Outcomes

#### 3.3.1. Psychological Outcomes: Depression and Anxiety Symptoms

**Figure 3:**
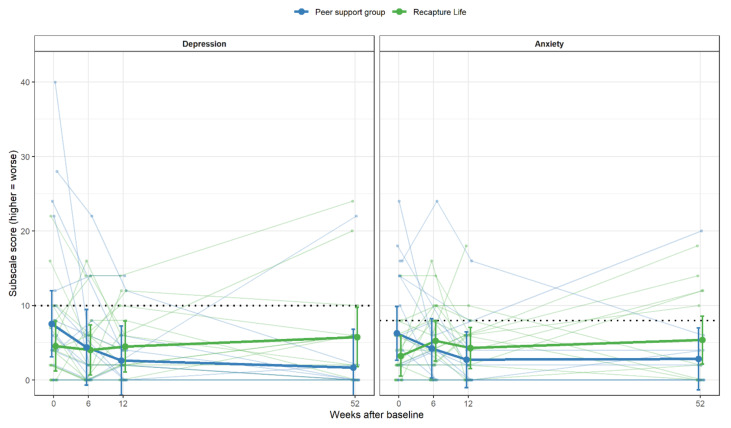
Depression and anxiety symptoms between Recapture Life and peer-support groups over time. Note. Individual scores are displayed, overlaid with thicker lines showing group-specific means and 95% confidence intervals. Dotted line indicates the upper end of ‘Normal’ range of symptoms on each subscale, with scores above this indicating Mild symptoms and above.

On average, participants across groups reported mean scores reflecting depression and anxiety symptoms in the normal range of the DASS-21 at each trial time point (Table 4). Three-way analyses showed no evidence that either Recapture Life or peer-support group participants differed to waitlist controls on depression or anxiety symptoms at the 6-week follow-up (*p’s* > 0.30). Focusing just on the two online interventions, adjusting for baseline, individuals allocated to Recapture Life had higher levels of anxiety at 12-weeks (*p* = 0.046), and 12-months post-intervention (*p* = 0.041), and higher levels of depression at 12-months (*p* = 0.041), compared to those in the peer-support group (Figure 3). There was no evidence that either intervention had differential effects according to an individual’s baseline depression or anxiety scores (*p*-values ≥ 0.092).

**Figure 4:**
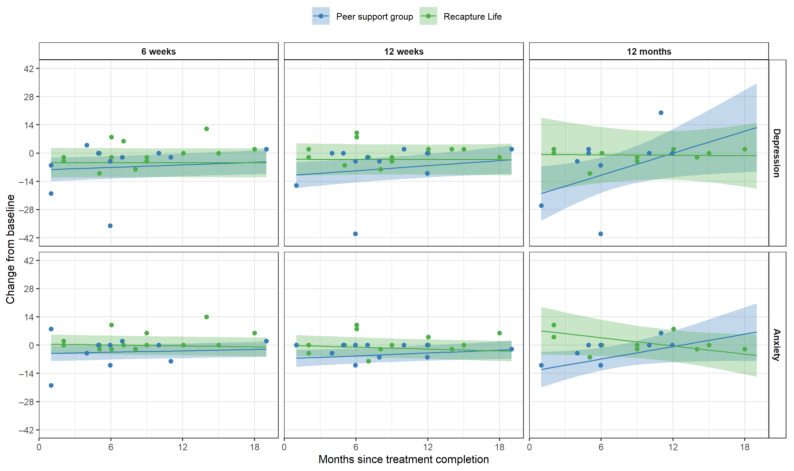
Between-group differences in change in depression and anxiety scores according to participants’ time since treatment-completion, across time-points. Note. Individual scores are displayed, overlaid with thicker lines showing group-specific means and 95% confidence intervals. Participants’ length of time since completing cancer treatment is represented along the X-axis (range: 0–18 months post-treatment); while each column of panels depicts data measured at different study time-points (6-weeks, 12-weeks, 12-months).

Post-hoc moderation analyses. Neither AYAs’ age nor sex moderated their scores on depression or anxiety in response to either intervention. However, evidence suggested that AYAs’ time since treatment-completion moderated the impact of the intervention on anxiety symptoms (*F_(_*_1*,*48*)*_ = 5.52, *p* = 0.023), but not depression (*p* = 0.10). These analyses suggested that the peer-support group intervention yielded better improvements in anxiety outcomes for AYAs closer to treatment completion, and that these participants gained more of this advantage over time (up to the 12-month follow-up). For AYAs further into survivorship post-treatment, Recapture Life appeared relatively more beneficial than the peer-support group in improving anxiety symptoms (Figure 4).

#### 3.3.2. Psychological Processes and Mechanisms: Cancer-Related Identity, Coping Skills and Family Functioning

##### Cancer-Related Identity Changes

Centrality of events. On average, AYAs’ scores indicated a high degree of illness centrality at each trial timepoint (Table 2). There was no evidence of between-group differences in the extent to which participants viewed their cancer experience as central to themselves/their lives, in either three-way or two-way analyses at any point (*p*-values ≥ 0.35).

Survivor identity scale. At baseline, most participants indicated that they leant towards the “survivor” end of the scale, with 87.2% scoring themselves higher than 5 (mean = 7.4, SD = 1.9; median = 7; range = 3–10). While Recapture Life participants identified more strongly as ‘cancer survivors’ at each follow-up timepoint than peer-support group control participants, there was no evidence of significant group differences over time (all *p*-values ≥ 0.55).

##### Coping Strategies

Coping skills use (KIDCOPE). Looking at the total number of different coping strategies used, there was no evidence of differences between the three groups immediately following the intervention (*p* = 0.24). On average, participants in both interventions reported increased use of coping strategies at the 6-week post-intervention follow-up, and then subsequently decreased their use of coping strategies at the later 12-week and 12-month follow-ups (Table 4). However, when comparing Recapture Life to the peer-support group over time, results indicated that Recapture Life participants reported using more coping strategies at 12-weeks than did peer-support group participants (OR: 2.35, CI: 1.03–5.37, *p* = 0.043). This pattern persisted with a similar effect size, though was no longer significant 12-months post-intervention (OR: 2.39, CI: 0.94–6.04; *p* = 0.066; Figure 4). From an average of 5.9 strategies used at baseline, this corresponded to a difference of 5.8 (RL) vs. 4.3 (PSG) strategies used at 12 weeks and 6.0 vs. 4.5 strategies at 12 months. Recapture Life participants also appeared somewhat (though not significantly) more likely than peer-support group participants to report that the coping strategies that they used were helpful at 6-weeks (OR: 2.05, CI: 0.89, 4.72, *p* = 0.091), 12-weeks (OR: 2.25, CI: 0.99–5.13, *p* = 0.053) and 12-months (OR: 2.37, CI: 0.97–5.81, *p* = 0.060; See Supplementary Data S6).

**Figure 5:**
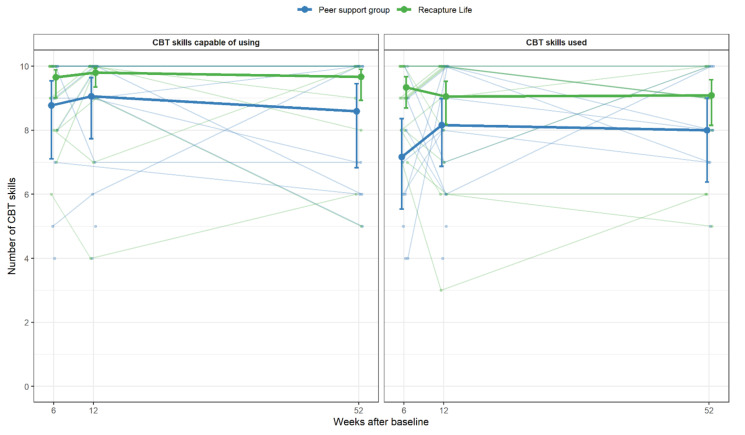
Cognitive-behavioral therapy skills confidence and use, by treatment arm. Note. Individual scores are displayed, overlaid with thicker lines showing group-specific means and 95% confidence intervals.

CBT skills. Overall, Recapture Life participants reported feeling capable of using a greater number of CBT skills, relative to peer-support group control participants (*OR*: 4.59, 95% CI: 1.19–17.76, *p* = 0.027; Table 2). The difference between groups did not appear to change over time (*p* = 0.92). In relation to participants’ self-reported *actual* use of CBT strategies, across each time point, Recapture Life participants reported ‘actually using’ a greater number of CBT strategies, relative to peer-support group controls. There was strong evidence that Recapture Life participants also used a higher number of CBT skills at the 6-week follow-up (OR: 5.58, 95% CI: 2.00, 15.56, *p* = 0.001; Table 4), with this difference lessening and becoming non-significant into the follow-up period (12-weeks: OR: 2.16, 95% CI: 0.78, 5.98, *p* = 0.14; 12-months: OR: 2.51, 95% CI: 0.78, 5.98, *p* = 0.12). Figure 5 depicts CBT skills confidence and use by treatment arm (Supplementary Data S7 depicts skills use according to individual CBT skill).

#### 3.3.3. Family Functioning

AYA outcomes. Across groups, AYAs’ mean scores on perceived family functioning indicated functioning in the adaptive range on average at almost all trial time-points. At the 12-month follow-up, only the communication subscale for the peer-support group control remained in the maladaptive/problematic functioning range (Table 2). Neither three-way analyses at 6-weeks (*p*-values ≥ 0.11), nor two-way analyses up to 12-months (*p*-values ≥ 0.10) showed any evidence of groupwise differences in any changes to general, problem-solving, or communication-related family functioning.

Support person outcomes. Three-way analyses indicated there was some evidence that the support people allocated to the peer-support group control reported more adaptive general family functioning relative to the waitlist group at the 6-week follow-up (*p* = 0.045), however no other significant effects of group or time emerged from either three- or two-way analyses.

Support person unmet cancer needs. At baseline, the top three high unmet needs (ranked as high/very high needs) by support person participants were all related to their worries, about ‘test results’ (11/16, 68.9%), ‘cancer returning’ (11/16, 68.9%), and whether ‘treatment worked’ (9/16, 56.3%). The number of perceived cancer-needs mostly declined over time for support people (Table 4) but there were no between-group differences on this over time (*p*-values > 0.10). Supplementary Data S8 presents baseline cancer-related needs for AYAs’ support people.

#### 3.3.4. Intervention Delivery: Fidelity, Group Cohesion, and Therapeutic Alliance

Manual fidelity. Reflecting the acceptability of Recapture Life, psychologists reported adhering to all session components in 82% of sessions, with no evidence of differences between Recapture Life and peer-support control online groups (*X*^2^(1) = 0.07, *p* = 0.80). Two cases of ‘manual deviations’ were reported, both in peer-support group sessions, involving unintended provision of coping skills. Treatment fidelity of recorded sessions, as rated by two independent raters, was also acceptable: Psychologists adhered to the manualized intervention components (specific to either Recapture Life or peer-support group control) in 97% of sessions and the blinded assessors correctly identified the intervention arm on 15/16 occasions (94%).

Therapeutic working alliance and group cohesion. Across the intervention period, AYAs in both Recapture Life (Week 2: M = 6.16 [SD = 0.98]; Week 6: M = 6.35 [SD = 0.64]) and peer-support group arms (Week 2: M = 6.19 [SD = 0.82]; Week 6: M = 6.06 [SD = 0.64]) rated therapeutic alliance with their group facilitator positively (on the 7-point scale). Likewise, group cohesion scores were positive across the follow-up period (Recapture Life: 6-weeks: M = 5.84 [SD = 0.62]; 12-weeks: M = 5.53, [SD = 0.83]; Peer-support group control: 6-weeks: M = 5.62 [SD = 0.85]; 12-weeks: M = 5.35, [SD = 0.93]). We observed no evidence of differences between Recapture Life and peer-support interventions in either the average score or change over time for working alliance (*p*-value = 0.80) or group cohesion (*p*-value = 0.70).

Perceived benefit and burden of intervention. (i) AYA participants. Across both Recapture Life and peer-support control groups, the majority of AYAs reported high perceived benefit at each time point, with a slight decline over time across both groups (*p* < 0.01). There were no between-group differences in perceived benefit over time (*p* = 0.93). The majority of AYAs also indicated low burden at all timepoints, with no evidence that this changed over time either between (*p* = 0.55), or across groups (*p* = 0.78). Table 4 depicts outcome measures across intervention arms. (ii) Support persons. Most support person participants reported low burden from involvement with the study (Table 2). Relative to the peer-support group control, more support persons allocated to Recapture Life reported deriving high personal benefit immediately following the online intervention (83.3% vs. 20.0% at 6 weeks). However, due to the small numbers, no further statistical tests were performed.

#### 3.3.5. Health Economic Analyses: Real-World Functioning, Health Service and Medication Use, and Costs

Real-World Functioning

Absenteeism: Days off work or study. AYAs’ self-reported absenteeism from paid work or school/university across the study period was highly variable (see Supplementary Data S9). There was no evidence to suggest that Recapture Life participants differed at any time-point to either peer-support group or waitlist controls in the number of days they took off from work or study across the timepoints, compared to baseline.

Days engaged with productive activities. There was a considerable degree of intra- and inter-personal variability in AYAs’ engagement with a range of productive activities across the study period (Supplementary Data S9). Although, again, there were no between-group differences in the mean number of days engaged in a range of productive activities over the past 28 days from baseline.

**Figure 6:**
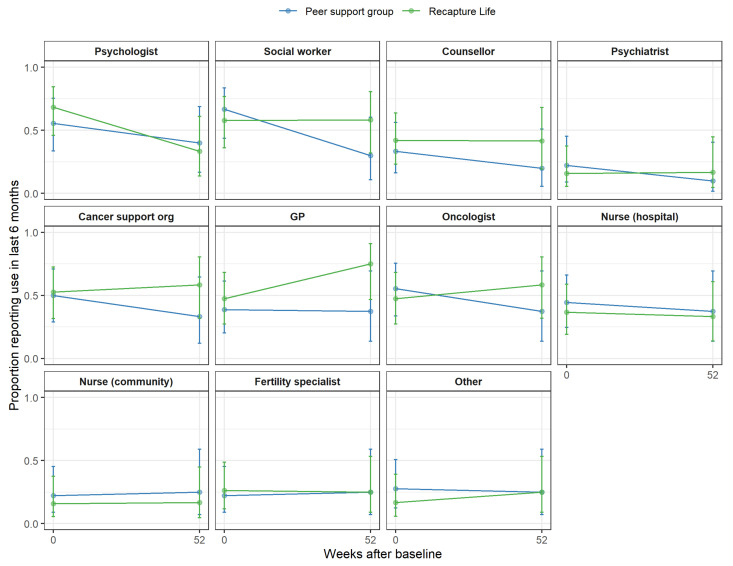
AYAs’ health service use over the past six months by healthcare professional type. Note. Individual scores are displayed, overlaid with thicker lines showing group-specific means and 95% confidence intervals.

Health and mental health service use. The intervention groups did not differ in health service use at baseline. When AYAs’ use of health services was examined, Recapture Life participants reported higher health service use (*p* = 0.05) between 6–12 months post-intervention compared to peer-support group control participants, after adjusting for reported use in the 6 months prior to baseline. There were no between-group differences on mental-health service use. Examined according to specific health-service type, the only significant finding was that Recapture Life participants reported accessing/seeing their GP significantly more often relative to the peer-support group at the 12-month follow-up (*p* = 0.015; see Figure 6).

There was some evidence that AYAs allocated to Recapture Life reported more Emergency Department visits at 12-months compared to peer-support group control, adjusted for baseline (with 23% Recapture Life participants (95% CI: 8–50%) compared with 0% of peer-support group (95% CI: 0–26%); Likelihood Ratio Test = 5.87; *p* = 0.015; depicted in Supplementary Data S10). No other between-group differences emerged in relation to hospital admissions over time.

Medication use. Over half of all participants in the waitlist (73%), peer-support (70%) and Recapture Life (56%) arms reported taking any medications at baseline. Two-way analyses comparing only the two online interventions indicated that while a higher proportion of the peer-support group controls reported taking medications at baseline relative to the Recapture Life group (72% compared to 58%) this pattern reversed somewhat over the intervention period (Supplementary Data S11). At baseline, psychotropic medication use was reported by 5 (28%) Recapture Life participants, 2 (18%) peer-support group participants, and no waitlist participants. At the 12-month post-intervention follow up, 5 (42%) Recapture Life participants continued to take psychotropic medication, compared to no peer-support group control participants. Due to small numbers, no statistical tests were performed.

Cost of delivery. Our modelling indicated that the cost of delivering Recapture Life ranged between an estimated AU$485–540 per participant. By contrast, the peer-support group control cost less to deliver, at an estimated AU$365–415 per participant.

Cost to AYAs and estimated travel costs saved. We estimated that on average, our participants avoided AU$260 in travel costs in terms of fuel costs alone, though there was a large range (SD = $342.26, range $8.95–$1124.09).

## 4. Discussion

Coined the ‘lost tribe’ in supportive cancer care [98], AYAs experience numerous barriers to accessing age-appropriate, specialist mental health support following cancer treatment [1,19,99]. The Recapture Life trial represents one of the first attempts, worldwide, to meet the psychological needs of AYA cancer survivors using innovative online technologies. Its tailored, manualized approach ensured that evidence-based content was reliably delivered with high fidelity, and modest costs. Our inclusion of an active, peer-support group control also enabled us to assess the benefits of online support provided by a peer group, as well as any additional or specific benefits of teaching evidence-based CBT skills.

Our data provided a mixed picture of the benefit of online psychological interventions, and specifically Recapture Life, at different time-points. We did not find improvements in our primary outcome (quality of life) over the intervention period. Indeed, contrary to our hypotheses, in the follow-up period, AYAs allocated to the Recapture Life intervention demonstrated increased perceptions of the negative impact of cancer. Similarly, while depression and anxiety scores remained in the normal (non-clinical) range across the intervention and study period, the data suggested that the Recapture Life group reported higher depression and anxiety symptoms at follow-up. When length of time since treatment was taken into account, AYAs closer to treatment completion appeared to garner the greatest benefit from non-directive peer-support-groups, in terms of anxiety symptom improvement, while AYAs further from treatment completion responded better to Recapture Life. Participants in Recapture Life also reported significantly greater self-efficacy and use of coping strategies relative to peer-support participants, although these coping-skill gains were not maintained by the 12-month follow-up.

It is possible that our quality of life evaluations of Recapture Life were susceptible to the documented psychometric phenomenon of ‘response shift’ [100]. Response shift involves individuals’ self-reported perceptions of their quality of life declining in response to a psychosocial intervention, which has heightened their level of insight and understanding around their distress and quality of life, as they integrate new understandings of how they are coping across domains. Our findings of increased perceptions of negative impact of cancer among the Recapture Life group may be consistent with this. Likewise, it is possible that the higher help-seeking from general practitioners, Emergency Department presentations and psychotropic medication use in the Recapture Life group similarly reflected a heightened awareness of the ongoing impact of cancer. As we did not build in measurements indexing response-shift, we cannot preclude this having had an impact on our quality of life-related outcomes. Recent longitudinal data from the BRIGHTLIGHT place of care study in the UK may echo this pattern; their data highlighted that AYAs receiving some or all of their care in hospital units specifically tailored for teenagers and young adults unexpectedly reported *poorer* quality of life over time, compared to AYAs who had received no care in such age-appropriate spaces [101]. The potential for the unintended, paradoxical effects of providing AYAs with peer- and cancer-related support warrants further study.

Recapture Life participants reported higher perceived negative impact of cancer, depression, and anxiety at follow-up; as such, it is possible that the skills-based model of group psychological intervention delivered through Recapture Life is not helpful for all AYA cancer survivors. Our data suggested, however, that AYA survivors may benefit from different types of online interventions at different points throughout the survivorship trajectory. Post-hoc analyses, taking into account the length of time since treatment-completion, suggested that our two intervention models may have been differentially beneficial for AYAs according to their passage through survivorship. Specifically, AYAs closer to treatment completion appeared to obtain more psychological benefits from the non-directive peer-support model of supportive discussion, while AYAs further into the post-treatment survivorship period appeared to derive more benefit from the CBT-based model accessed through Recapture Life. This finding warrants further investigation.

The finding that AYAs closer to treatment completion appeared to benefit the most from the non-directive peer-support model is consistent with extensive literature documenting AYAs’ unmet needs for peer-support [11,102,103] and perceptions of social isolation and feeling ‘different’ being key sources of distress [104,105]. Unlike Recapture Life, where CBT coping strategies were taught and discussed in a structured way, the peer-support group afforded more time for open-ended, non-directive peer-to-peer conversation. It may be that AYAs further from cancer treatment completion benefited more from learning CBT-based coping skills due to the increased distance gained over time from their acute hospital experiences, which may have enabled greater reflection on their cancer experiences. It is also possible that in the intervening period since finishing treatment, AYAs further into survivorship may have had time to re-integrate themselves more into routine life post-treatment, including study, work, and social relationships. AYAs further into survivorship may also simply have had more time to recover physically and cognitively, potentially enabling greater capacity to actively engage with CBT strategies. This time and experience may influence AYAs’ need and motivation for learning new, adaptive coping strategies, particularly if they find that coping strategies that served them well during the active treatment period become less adaptive over time. Our trial used random allocation, and we did not assess participants’ baseline degree of motivation, expectations, or preferences for different types of interventions. Consequently, it is unclear whether treatment preferences, motivation, or readiness may have played any role in the extent to which AYAs benefited from the two models of online support.

The selective-prevention approach used in Recapture Life aimed to equip AYAs with coping strategies to bolster quality of life at a known point of risk (cancer treatment completion and early survivorship). In the years since we developed Recapture Life, large-scale reviews have suggested that, while there may be some evidence to support the efficacy of selective-prevention interventions in reducing the severity of mental health disorders in the short term, longer-term data is more mixed, as is data on the efficacy of selective-preventative interventions in preventing the onset of mental health disorders [9,10]. Our data indicate that those who took part remained highly-engaged throughout the intervention, increased their coping skills confidence and use, and qualitatively reported benefits [36]. This directly addresses a gap identified by AYA survivors in post-treatment supportive care [106]. However, given that our sample was not clinically-distressed, it may not be representative of the diversity of AYA survivors. Consequently, what was intended as a selective-prevention intervention approach (teaching coping strategies to at-risk, distressed AYAs) may have inadvertently become a universal-prevention approach (whereby we taught coping strategies to individuals who were overall not distressed), by virtue of the individuals who chose to participate.

Related to this, it is worth considering whether the demands of the multi-session intervention, together with the time and organizational skills required to participate in the trial (with multiple questionnaires across a 12-month period) may have similarly skewed our cohort towards more high-functioning individuals. Although randomized-controlled trials are considered the methodological gold-standard, researchers have also highlighted how the rigors of the recruitment processes and study designs can inadvertently impact the extent to which study findings can be generalized to the real world, over and above the role of the eligibility criteria [107]. Future research using more diverse research designs, more closely approximating how individuals select and access support in real-world settings is needed to complement more tightly-controlled trial designs, such as the one we used.

Although as a group, AYA cancer survivors share unique mental health risks and developmental vulnerabilities, our data echoes previous reports highlighting the considerable variability in survivors’ functioning and psychological needs [1,87]. For example, our data on AYAs’ real-world functioning across the study period highlighted the extent to which AYAs were functioning differently at different timepoints as they progressed further into survivorship. The notion that survivorship is a dynamically changing period—as opposed to a uniform state of adjustment post cancer-treatment—is not new [108]. For AYAs, survivorship is likely to involve multiple, interacting trajectories that could impact young people’s mental health. For example, alongside the normal developmental trajectories of socio-emotional development and age-related mental health risks, AYA cancer survivors’ cancer-related psychological processing is likely to be evolving as they reflect on their cancer experiences and related identity changes [18]. Additionally, as AYAs become increasingly re-integrated into life activities, they may experience the re-emergence of non-cancer-related life stressors alongside positive changes. Amidst these intersecting survivorship demands, the right time to intervene remains unclear. Understanding what kinds of support AYAs need, and when, are crucial questions that remain unanswered.

### 4.1. Limitations

The lessons learned through this trial need to be placed in the context of several limitations. Due to recruitment challenges, our trial was ultimately under-powered to detect clinically-meaningful differences in our primary outcome measure, quality of life. The significant delays we experienced in our trial to receiving ethical approval across all sites (median of 16 weeks to approval, range: 4–39 weeks) [36,94] had a cumulative flow-on effect on the overall number of AYAs ultimately approached. While our attrition rates were lower than anticipated, our response rates fell well below expectations (~30%). Larger studies are needed to generate more conclusive data. Yet, there is growing evidence about the recruitment challenges faced by researchers testing online mental health interventions [31,109,110,111,112], with one study recruiting only 10 AYA cancer survivors out of 213 potential eligible participants contacted (4.7%) [113]. Reviews have highlighted that randomized psychosocial studies average lower opt-in rates in pediatric psycho-oncology (45.5%) relative to studies examining health behaviors (78.2%) or neurocognitive functioning (91%); group-based interventions also tend to yield lower opt-in rates [114]. Recruitment to pediatric psycho-oncology studies also appears more successful when face-to-face recruitment methods are used (as opposed to the letter invitation method we used), and when recruiting closer to diagnosis [115].

Our choice of quality of life as the primary outcome measure may also have been misaligned with the CBT skills-based processes targeted within our intervention. Among psychometric measures validated for AYAs, there is a predominance of quality of life measures [116], which are also valued within the field of health economics and prioritized by research funding bodies. While important, quality of life is also acknowledged to be a broad outcome and challenging to improve [116,117,118]. For AYAs in the early phases of cancer survivorship, other life events may have been more powerful than the impact of our six-week intervention in influencing their quality of life. In terms of measurement, focusing primarily on assessing common mental disorders, together with other factors important for AYAs’ resilience (such as social support) may have been better.

Participants’ medical risk across this early survivorship period is a relevant consideration within this trial, which included participants who had completed cancer treatment with curative intent and achieved remission. In retrospect, this likely resulted in treating our sample as more uniform than it may have been, in terms of short- versus long-term risk of relapse. Our experiences documented here, and previously [119], in terms of the relatively high number of participants who relapsed during the trial underscores the important distinction between achieving short-term remission compared with the prospect of a long-term ‘cure’. Indeed, that five (12.5%) of our total sample have died since trial intake highlights that even after ‘successful’ completion of cancer treatment, many of these AYAs were contending with serious health concerns. Due to our small sample, it was not possible to examine the impact of these medical factors (which varied somewhat across trial arms) on psychological outcomes with any further granularity. Whether skills-based interventions such as Recapture Life meet the psychological needs of AYAs with varying diagnoses and prognoses in survivorship therefore remains unclear.

There are several implications of these medical risks. Firstly, it is possible that variables that we did not measure—such as fear of cancer recurrence, or post-traumatic stress symptoms—may have explained the observed increases in perceived negative impact of cancer, depression, and anxiety. Recent research has highlighted the prevalence of fear of recurrence for AYA cancer survivors and the impacts of this on long-term adjustment [120]; it is possible that any AYA participants who were experiencing either clinically-significant fear of cancer recurrence, or post-traumatic stress symptoms, may not have found Recapture Life to adequately address this. Secondly, in this context, the dosage or content of Recapture Life may have been insufficient to mitigate these cancer-related stressors for some survivors, over and above the benefits of non-directive discussion and peer support. Assessing the appropriateness of survivorship interventions for AYA cancer survivors across the spectrum of prognostic outlooks is a challenge for the field and an important area for future research [119].

Our relatively small number of support person participants constrained our ability to quantify, either the benefit of the intervention to them, or the impact of their inclusion for AYAs and the family system (through broader dissemination of coping skills through AYAs’ support networks). Parents and caregivers play an important role in assisting AYAs to negotiate the disruption caused by cancer [121,122], and their inclusion in skills-based programs appears to enhance AYAs’ own psychological outcomes [19]. However, with the intention of not undermining AYA participants’ autonomy, we made the decision in this trial to make support person participation optional. This design resulted in greater statistical complexity, as well as a smaller support person sample which limited our ability to delineate the impact of support person involvement in Recapture Life. Finally, waitlist controls are also now acknowledged to have unintended adverse effects on participants who experience them [123]. It is unclear whether and how this may have influenced our present findings, including waitlisted AYAs’ subsequent experiences of the trial or either online intervention.

### 4.2. Future Directions

Our study contributes to a growing evidence base regarding online psychological interventions for AYAs living with cancer, which has been of particular interest during the global COVID-19 pandemic [22,23]. Even prior to the COVID-19 pandemic, online psychological interventions have demonstrated acceptability, feasibility, and safety with increasingly rigorous data for AYAs [21]. Beyond COVID-19, the acceptability patients have with telehealth interventions may be even greater. However, a remaining gap in the literature is determining what psychological intervention models are most suitable, desirable, beneficial, and at what times along the AYA cancer trajectory. Our data here and previously [36] accords with other studies in supporting the acceptability of peer-group based online interventions for those AYAs who take part [33,36], and also echo other data highlighting that not all AYAs wish to participate in formal peer-support programs with cancer survivors [122]. However, our peer-support group control clearly conferred some benefits; this may accord with recent research highlighting the potential for peer-support, including connections with AYA cancer survivors, to promote post-traumatic growth (something we did not measure) [124]. Research is needed to understand, not only the points at which AYAs are likely to gain most benefit from different forms of intervention, the relative benefits of structured versus unstructured peer-support interventions, the ideal composition for peer-group based interventions, the appropriateness of each support model for AYAs with varying degrees of distress, and importantly, when AYAs are most likely to take up these opportunities. Other research evaluating parent- and family-based interventions in oncology has indicated that perceived need, acceptability, and intervention uptake are often unrelated, and while families may express wanting interventions at a certain points of crisis (e.g., diagnosis), this may not translate into intervention uptake when given the opportunity [125,126]. Our data may indicate that AYAs gain greater benefit from structured, skills-based programs further into survivorship—a proposition requiring further study. Further, in-depth qualitative as well as quantitative data may illuminate what drives AYAs’ choices around different types of support-seeking, and the benefits they gain from this.

Our findings on cost indicate that although online programs may be deliverable at modest per-participant costs, they still involve considerable time and resources to deliver. It remains unclear what level of ‘cost’ versus ‘benefit’ may be acceptable, and feasible, within public health contexts, as well as in not-for-profit, community-based settings where much psychosocial support in cancer survivorship is provided. Although health economic data highlights the considerable social burden of AYA cancer in terms of the quality of life years lost [127], little data has quantified the social cost of the mental health impacts of AYA cancer. Given the many life-years that stand to be gained from curative cancer treatment, it seems reasonable to expect that preventing mental health issues among AYA survivors would be associated with reduced social costs. Additionally, as newer models of survivorship support continue to be developed and evaluated internationally, it may be useful to evaluate AYA survivors’ preferences for, and the relative value they place on different models of care. Health economics methodologies such as discrete choice experiments may be particularly useful in advancing knowledge on this issue.

Beyond AYAs’ preferences for support models, the question remains as to which model(s) of mental health support may be most effective and appropriate in cancer survivorship, as mental health concerns among AYA cancer survivors are prevalent [1,2], and are a recognized unmet need [6]. Reconciling these issues to develop scalable evidence-based models of mental health assessment, and intervention, throughout cancer survivorship poses a considerable challenge to the field. It is clear from our study and others that challenges remain in understanding how best to engage AYAs with evidence-based support. Overcoming the many barriers to seeking and accessing care for AYAs is likely to require better partnerships across the health system, including with primary care (such as general practitioners).

More broadly, the development and implementation of psychological interventions, such as the CBT-based intervention tested here, takes the approach of targeting modifiable factors at the individual level. While important, this individually-oriented approach may overlook broader contextual and social factors that are critical for good mental health at the population level [128]. There is increasing acknowledgement that the absence of mental disorders is not necessarily the same as having good mental health [9]. To achieve the latter, addressing the social determinants of mental health in a truly preventative way appears critical. For AYA cancer survivors, this may include proactively addressing key factors related to their ongoing social connections, smooth re-integration with family and communities, and sustained, supported engagement with education and work during and beyond cancer treatment—factors known to be linked to AYAs’ mental health [129,130]. Future models of mental health support for AYA cancer survivors in clinical practice will need to better integrate preventative and targeted approaches to balance the dual goals of supporting good mental health of the population as a whole, whilst still addressing mental disorders of a subset in a targeted way. Advancing the field will require us to better understand the optimal strategies to use to achieve each goal.

## 5. Conclusions

This trial demonstrated that AYAs engaged well with online, supportive interventions in the first two years following cancer treatment, though recruitment was a major challenge. We did not find a positive impact of Recapture Life on quality of life, assessed as perceived impacts of cancer in the short term, which may reflect the impact of recruitment on study power. AYAs who participated in Recapture Life reported higher perceived negative impacts of cancer, anxiety, and depression at follow-up compared to a peer-support group control. Our data suggest that AYA cancer survivors may respond differently to different models of online support according to how recently they completed treatment. Understanding how best to engage AYA cancer survivors in psychological support, and at which points in time, remains a challenge for the field.

## Figures and Tables

**Table 1:** Recapture Life and peer-support control group weekly content delivered by the psychologist during each module.

Module		Recapture Life Program (Skills Focus)	Peer-Support Group (Discussion Topic)
1	*“What just happened to me??” (being a young person after cancer)*	Psycho-education & normalization: Discussion of range of common emotional and cognitive responses to cancer for individual and family. Building rapport and a safe, trusting group environment.	The cancer experience and coming off treatment. Common emotional responses to cancer for individual and family.
2	*Getting back into the swing of things after cancer*	Healthy balanced lives & behavioral activation: Discussion of impact of cancer on all areas of life including exercise, hobbies. Use of ‘ACE’ activity scheduling ^a^ to tackle ‘inactivity trap’ and help self-esteem/stress. Positive activities scheduling to improve mood and increase sense of control.	Impact of cancer on hobbies and lifestyles. Changes to routines, hobbies and activites.
3	*How has cancer changed the way I think?*	ABCD model & thought challenging: Introduction to ABCD model ^b^ and idea of ‘unhelpful thinking styles’. Cognitive challenging. Identifying underlying beliefs about their cancer experience, self and future.	How has cancer changed my family? Family reactions across the cancer trajectory, positive/challenging family supports.
4	*The ‘elephant in the room’: Thinking about the cancer coming back*	Acceptance-based strategies: Evaluating the usefulness of certain thoughts; thought suppression experiment; using worry postponement and other behavioral strategies to manage ‘questions without answers’.	The big, ‘scary’ stuff: Niggling thoughts about illness, death and dying. Discussing/normalizing existential and illness concerns.
5	*Talking all things cancer: Simple communication skills for difficult situations*	Social support: Seeking out social support; managing unhelpful/difficult thoughts around friends/relationships; assertive communication skills. Strategies to reconnect with old friends and develop new friendships and relationships.	Talking all things cancer and friends. Common social/friendship issues across the cancer trajectory; difficult topics to raise.
6	*Goal-setting and planning for the future (even when things feel up in the air)*	Goal setting: Applying reappraisal and problem-solving skills to the future to develop realistic post-cancer goals. Psychological ‘relapse’ prevention: Anticipating future difficult situations and reviewing skills learnt to manage these situations in the future.	Moving on: Looking ahead to the future. Normalizing uncertainty/change; discussing potential ‘positives’; things to look forward towards.

Participants were aware of the topic heading/focus for each module. Tailored supportive counselling [57] was used in all sessions and was common to both the Recapture Life and peer support group interventions, and involves empathic listening to normalize the range of AYA experiences and promote peer discussion/support. ^a^ ACE = Achievement, Connectedness, Enjoyment, an acronym designed to help individuals’ consider what function different activities in their life serve for the purposes of stimulating behavioral activation, pleasant activity scheduling and balance. ^b^ ABCD model refers to the cognitive-behavioral model, whereby, individuals can understand their emotional responses to situations through considering the A=‘Activating event’ or situation; the resulting B=‘Beliefs’ or automatic thoughts that followed, the C=‘Consequences’ in terms of emotions and physiological sensations, and then the D=‘Doing’ actions (behaviors) that they engaged in as a result.

**Table 2:** Assessment schedule for the Recapture Life study.

Domain Assessed	Measure and Subscale Information	Scoring and Analysis Information	Psychometric Validity Data Available	Timepoint Administered				
				Intake	T1 ^a^	During Intervention ^b^	T2 ^c^	T3 ^d^
Baseline characteristics	Psychosocial Adjustment to Illness Scale-Interview form (PAIS) ^e^	N/A	-	X	-	-	-	-
	Demographic data * (AYAs’ age, sex, level of educational attainment, employment status, family structure, diagnosis, treatment regimen) including six items from the Intensity of Treatment Rating Scale [68]	N/A	-	-	X	-	-	-
Quality of life	Impact of Cancer Scale (IOCS): five subscales used included Social life (negative), Uncertainties, worries and wonders (negative), Sense of purpose/goals (positive), Identity (positive), Health behaviors (positive). ^f^ Due to the younger age range of Australian AYAs relative to the US validation cohort, in consultation with the scale’s developer, we included 55 of the original 91 items [69].	0 = strongly disagree) to 4 = strongly agree). Following the method used by Zebrack and Landier, [70] we calculated overall positive and negative impacts by summing items from positive and negative subscales respectively.	Validated in AYAs with cancer aged 18–39, good construct and concurrent validity, and test-retest reliability [58,71]	-	X	-	X	X
Psychological outcomes	Depression, Anxiety, Stress Scales-short form (DASS-21): depression (7 item) and anxiety (7 item) subscales	4-point scale, rating extent to which they had experienced each symptom in the past week (1 = “Not at all” to 4 = “Most of the time”).	Australian adolescents [72] cancer patients [73] strong internal consistency and reliability [72,74]	X	X	-	X	X
Psychological mechanisms	Centrality of Events Scale-Short Form	5-point Likert scale to questions relating to their cancer experience as a whole. (range: 7–35)	UK Young people aged 8–18 with cancer [75] Version of CES modified for cancer survivors age 13–23 [76]	-	X	-	X	X
	Perception as “cancer survivor” item (study-developed)	10-point scale: 1 = patient; 10 = survivor	-	X	X	-	X	X
	McMaster Family Assessment Device *—We administered the family communication (6 items), problem-solving (5 items), and general functioning (12 items) subscales in Recapture Life.	Each item uses a Likert scale scored from 1 to 4, with the subscale score calculated as the average of the item scores, and higher scores indicating more problematic functioning.	US Adolescents (13–19) currently undergoing treatment [77] US adolescents (11–19) post-treatment [78]	-	X	-	X	X
	KIDCOPE-Older Version: Respondents name a recent cancer-related problem and rate 8 coping strategies for frequency of use (“Did you do this?”), and efficacy (“Did it help?”). Measures positive (e.g., social support, cognitive restructuring) and negative (e.g., resignation, social withdrawal) adolescent coping approaches	Frequency was measured as a binary response (“Yes”/“No”) and efficacy was measured on a 3-point scale (“Not at all”/“A little”/“A lot”).	US adolescents (12–18) with cancer [79]	-	X	-	X	X
	Cognitive-behavior therapy (CBT) skills (study-developed): Assessing participants’ acquisition of CBT skills, e.g., identifying thoughts/feelings in response to cancer treatment, and recognizing circular ruminative thinking processes	Participants were asked to rate their confidence (“since the online group program sessions, … did you feel like you could…” (Yes/No)) and their actual use of each skill (“did you actually do…?” (Not at all/A little/A lot))	-	-	X	-	X	X
Support person outcomes	Cancer Needs Questionnaire for Parents/Carers (CNQ-PC) ^: 17 items ^g^ addressed their relationship with the AYA, their ability to communicate, changes in relationships and friendships, and worries about the AYA’s cancer returning. These cancer needs were addressed in the Recapture Life support person emails.	5-point rating scale, with options ranging from “no need” to “very high need”	-	-	X	-	X	X
Intervention delivery factors	Homework Compliance Scale [80] ^e^	N/A	N/A	-	-	X	-	-
	Emotion thermometers tool *^,^^e^	N/A	N/A	X	-	X	X	X
	Working Alliance Inventory-Short Form: four items ^h^	7-point scale, 1 = ‘doesn’t correspond at all’ to 7 = ‘corresponds exactly’	AYAs as young as 11 years [81,82].	-	-	X	-	-
	California Psychotherapy Alliance Scale-Group (CALPAS-G): four items ^i^	0 = ‘not at all’, to 6 = ‘very much so’	-	-	-	X	X	-
	Benefit/burden of intervention *	5-point rating scale, “Not at all” to “Very much”	Hospital patients age 18–21 [67].	-	X	X	X	X
Health economics	Absenteeism from study/work	Estimated days absent over the past 4 weeks	-	-	X	-	X	X
	Engagement with productive activities: including ‘Paid work of any kind’, ‘Study or learning of any kind (school, university, TAFE, other courses)’, ‘Exercise or sports’, ‘Personal hobbies (e.g., art, music, films, books, outdoor activities, cooking)’, ‘Socializing with friends’, and ‘Socializing with other young people with cancer (includes connecting online)’. (study-developed)	Estimated days engaged in any of these productive activities over the past 4 weeks	-	-	X	-	X	X
	Health service use: General health services included visiting a general practitioner, oncologist/radiation oncologist, nurse in hospital, nurse in community, or fertility specialist. We also asked whether participants had any emergency department visits or hospital admissions. Mental health services included visiting a psychologist, social worker, counselor, or psychiatrist, as well as community-based cancer support organizations.	For the purposes of our analysis, participants’ health services use was assessed according to frequency of use (not cost) by profession, as well as across total, general, and mental health service use categories.	-	-	X	-	X	X
	Medication use: Participants reported whether they were currently taking any medications/supplements, and to indicate the reason for their use over the past week, the past four weeks, and the past six months. The classification of these medications was subsequently manually checked by a senior pediatric oncologist (RC), with reference to the Monthly Index of Medical Specialties online database.	Use was reported according to the number and classification of medications (not cost).	-	-	X	-	X	X

Intake = comprised a telephone interview to determine study eligibility and screen for mental health risk factors and distress that would preclude participation, as well as administer the PAIS interview. ^a^ T1 = Baseline; ^b^ During intervention = weekly prior to intervention sessions 2–6; ^c^ T2 = post-intervention; ^d^ T3 = 12-month follow-up. ^e^ Data published elsewhere: PAIS qualitative data [83,84,85,86,87] Homework compliance and emotion thermometers tool data [36] ^f^ IOCS: For our Australian AYA sample, for example, some items, e.g., those relating to financial concerns, were less relevant. ^g^ CNQ-PC items: a subset of 17 items were chosen to specifically map onto concepts addressed through support-person materials in the Recapture Life intervention including 8 items related to ‘Worrying about…’, 4 items related to ‘Coping with…’, and 5 items related to ‘Knowing how to…’ (see also Supplementary Data S8) ^h^ Working Alliance Inventory items represented 3 conceptual factors, Goal of treatment (e.g., ‘My group leader and I agree on what is important for me to work on’), Task (e.g., ‘I believe that the way we are working with my concerns is correct’), and Bond between therapist-client (e.g., ‘I feel that my group leader appreciates me’). Participants were informed the psychologist would not see their ratings. ^i^ Items indexed the factors of Patient Working Capacity, Patient Commitment, and Member Understanding and Involvement from the overall scale. * Measures with an asterisk also used in support-person participants at these same time-points. ^ Measure given only to support-person participants.

**Table 3:** Participant demographics by trial arm.

	Waitlist (*n* = 11)	PSG (*n* = 10)	RL (*n* = 19)	Total (*N* = 40)
**Sex**				
Male, n (%)	6 (55)	5 (50)	8 (42)	19 (48)
Female, n (%)	5 (45)	5 (50)	11 (58)	21 (52)
**Participant age (*n* = 38) ^**				
Mean (SD)	20.9 (3.1) ^	22.5 (2.5)	19.4 (2.6) ^	20.6 (3.0) ^
Median (IQR)	20.0 (18.2, 23.8) ^	23.0 (20.8, 23.8)	19.0 (18.0, 20.0) ^	20.0 (18.0, 23.0) ^
Range	17–26	18–26	15–25	15–26
**Highest education attained**				
Year 10 or below, n (%)	2 (18)	0 (0)	4 (21)	6 (15)
Year 12, n (%)	3 (27)	5 (50)	10 (53)	18 (45)
Apprenticeship, n (%)	0 (0)	1 (10)	2 (11)	3 (8)
TAFE or certificate/diploma, college, n (%)	2 (18)	1 (10)	1 (5)	4 (10)
University degree, n (%)	4 (36)	3 (30)	1 (5)	8 (20)
**Participant employment status**				
Employed: Full-time, part-time or casual, n (%)	6 (55)	7 (70)	8 (42)	21 (53)
Unemployed: Student, n (%)	2 (18)	1 (10)	7 (37)	10 (25)
Unemployed: Non-student, n (%)	3 (27)	2 (20)	3 (16)	8 (20)
**Distance from nearest capital city (km)**				
Mean (SD)	113 (177)	71 (90)	69 (106)	82 (124)
Median (IQR)	11 (8, 207)	20 (10, 102)	18 (9, 78)	17 (8, 97)
Range	3.8–389	5.2–275	4.9–429	3.8–429
ARIA classification ^1^				
Major city, n (%)	8 (73)	8 (80)	12 (63)	28 (70)
Inner regional, n (%)	3 (27)	1 (10)	5 (26)	9 (22)
Outer regional, n (%)	0 (0)	1 (10)	2 (11)	3 (8)
**Aboriginal and/or Torres Strait Islander**				
No, n (%)	10 (91)	10 (100)	18 (95) ^	38 (95) ^
Yes, Aboriginal, n (%)	1 (9)	0 (0)	0 (0) ^	1 (2) ^
**Speaks language(s) other than English at home**				
No, n (%)	9 (82)	10 (100)	15 (79) ^	34 (85) ^
Yes, n (%)	2 (18)	0 (0)	3 (16) ^	5 (12) ^
**Country of birth**				
Australia, n (%)	9 (82)	10 (100)	17 (89)	36 (90)
Other, n (%)	2 (18)	0 (0)	2 (11)	4 (10)
**Age at cancer diagnosis**				
Mean (SD)	19.4 (4.0)	21.2 (2.9)	17.8 (2.4)	19.1 (3.3)
Median (IQR)	20.0 (17.0, 22.5)	21.5 (19.5, 22.8)	17.5 (16.2, 19.5)	18.0 (17.0, 21.5)
Range	11–25	16–25	13–23	11–25
**Cancer type**				
Blood, n (%)	6 (55)	4 (40)	10 (53)	20 (50)
Solid tumor, n (%)	4 (36)	4 (40)	9 (47)	17 (42)
Brain, n (%)	1 (9)	2 (20)	0 (0)	3 (8)
**Cancer stage at diagnosis**				
Stage 1, n (%)	1 (9)	4 (40)	1 (5)	6 (15)
Stage 2, n (%)	3 (27)	2 (20)	3 (16)	8 (20)
Stage 3, n (%)	1 (9)	1 (10)	2 (11)	4 (10)
Stage 4, n (%)	4 (36)	1 (10)	3 (16)	8 (20)
Unsure, n (%)	1 (9)	1 (10)	7 (37)	9 (22)
**Cancer risk level**				
Standard, n (%)	2 (18)	0 (0)	3 (16)	5 (12)
Low, n (%)	2 (18)	1 (10)	0 (0)	3 (8)
Intermediate, n (%)	2 (18)	2 (20)	5 (26)	9 (22)
High, n (%)	2 (18)	2 (20)	6 (32)	10 (25)
Unsure, n (%)	3 (27)	5 (50)	4 (21)	12 (30)
**Treatment(s) received**				
Surgery, n (%)	8 (73)	7 (70)	10 (53)	25 (62)
Chemotherapy, n (%)	10 (91)	8 (80)	17 (89)	35 (88)
Radiotherapy, n (%)	6 (55)	2 (20)	5 (26)	13 (32)
BMT, n (%)	1 (9)	1 (10)	4 (21)	6 (15)
**Intensity of Treatment Rating ^2^**				
1, n (%)	0 (0)	1 (10)	1 (5)	2 (5)
2, n (%)	4 (36)	3 (30)	5 (26)	12 (30)
3, n (%)	7 (64)	5 (50)	10 (53)	22 (55)
4, n (%)	0 (0)	0 (0)	2 (11)	2 (5)
(Missing), n (%)	0 (0)	1 (10)	1 (5)	2 (5)
**Months since treatment completion**				
Mean (SD)	8.0 (5.5)	6.4 (4.0)	9.1 (4.7)	8.0 (4.7)
Median (IQR)	6 (6, 9)	6 (4, 10)	9 (6, 12)	7 (5, 12)
Range	2–19	1–12	2–18	1–19
**Disease progression**				
On-trial relapses, n (%)	-	2 (11.1)	2 (12.5) *	4 (11.7)
Overall relapses, n (%)	-	3 (18.8) **	6 (40.0) ***	9 (29)
Deaths ^3^	-	1 (10)	4 (21)	5 (12.5)
**Self-rated health**				
Excellent, n (%)	3 (27)	0 (0)	3 (16)	6 (15)
Very good, n (%)	3 (27)	4 (40)	7 (37)	14 (35)
Good, n (%)	2 (18)	5 (50)	5 (26)	12 (30)
Fair, n (%)	3 (27)	1 (10)	3 (16)	7 (18)
Poor, n (%)	0 (0)	0 (0)	0 (0)	0 (0)
Unknown, n (%)	0 (0)	0 (0)	1 (5)	1 (2)
**Pre-diagnosis mental health service use ^4^**				
Yes, n (%)	3 (27)	4 (40)	10 (53)	17 (42)
No, n (%)	8 (73)	6 (60)	8 (42)	22 (55)
(Missing), n (%)	0 (0)	0 (0)	1 (5)	1 (2)
**Parents’ marital status**				
Separated or divorced, n (%)	5 (45)	5 (50)	7 (37)	17 (42)
Not separated or divorced, n (%)	6 (55)	5 (50)	11 (58)	22 (55)
**Support person relationship**				
Mother, n (%)	4 (36)	4 (40)	4 (21)	12 (30)
Father, n (%)	0 (0)	0 (0)	1 (5)	1 (2)
Spouse/Partner, n (%)	1 (9)	2 (20)	0 (0)	3 (8)
Other/Unknown, n (%)	0 (0)	1 (10)	1 (5)	2 (5)
(No support person), n (%)	6 (55)	3 (30)	13 (68)	22 (55)
**Support person age ^^**				
Mean (SD)	46.4 (13.3)	38.0 (12.9)	49.8 (4.8)	43.9 (11.9)
Range	24–59	21–53	43–56	21–59

RL = Recapture Life, PSG = Peer-support group, BMT = Bone marrow transplant, SD = Standard deviation; IQR = Inter-quartile range. ^ denotes some variables missing for some participants due to a missing response for some items. ^^ denotes some variables missing for some items. * of 16; n = 4 missing. ** of 16, n = 2 missing. *** of 15, n = 5 missing. ^1^ The Accessibility/Remoteness Index of Australia (ARIA) is a standardized classification and index of remoteness from service centers [95]. ^2^ The Intensity of Treatment Rating (ITR-3) is a reliable and valid scale for classifying pediatric oncology treatment protocols [68]. ^3^ Of these deaths, four were related to cancer and one was unrelated. ^4^ From psychologists, psychiatrists, counsellors or social workers.

**Table 4:** Outcome measures * by intervention arm across timepoints.

		6 Weeks	12 Weeks	12 Months
AYA Outcomes				
High perceived benefit ^a^–n (%)	PSG	-	8 (53.3)	7 (64.0)
	RL	-	12 (67.0)	9 (75.0)
Low burden ‡–n (%)	PSG	-	14 (93.3)	10 (91.0)
	RL	-	16 (88.9)	10 (83.3)
**Psychological outcomes**				
DASS-21 Depression^1^ M (95% CI)	PSG	4.4 (−0.7, +9.5)	2.6 (−2.0, 7.3)	1.7 (−3.5, 6.8)
	RL	4.0 (0.7, 7.4)	4.5 (1.1, 7.9)	5.8 (1.8, 9.7)
DASS-21 Anxiety ^1^M (95% CI)	PSG	4.3 (0.3, 8.2)	2.7 (−1.0, 6.5)	2.8 (−1.3, 7.0)
	RL	5.3 (2.6, 8.0)	4.3 (1.5, 7.1)	5.4 (2.2, 8.6)
**Mechanisms and process variables**				
Centrality of Events ^	PSG	26.09 (23.41, 28.78)	26.02 (23.48, 28.56)	27.99 (25.22, 30.77)
	RL	26.28 (24.44, 28.12)	25.38 (23.51, 27.25)	27.48 (25.37, 29.60)
Survivor label	PSG	6.4 (5.0, 7.9)	7.3 (6.1, 8.6)	6.9 (5.4, 8.3)
	RL	7.5 (6.6, 8.4)	8.2 (7.4, 9.1)	8.0 (7.0, 9.0)
KIDCOPE—strategies used	PSG	5.8 (4.6, 6.6)	4.3 (3.2, 5.3)	4.5 (3.2, 5.6)
	RL	6.3 (5.7, 6.8)	5.8 (5.1, 6.5)	6.0 (5.1, 6.7)
KIDCOPE—strategies that helped	PSG	5.0 (3.9, 6.0)	4.4 (3.3, 5.4)	4.1 (2.9, 5.3)
	RL	5.7 (5.0, 6.3)	5.3 (4.6, 5.9)	5.2 (4.3, 5.9)
CBT skills—confidence, M (95% CI)	PSG	8.8 (7.1 9.5)	9.1 (7.7, 9.6)	8.6 (6.8, 9.5)
	RL	9.7 (9.0, 9.9)	9.8 (9.4, 9.9)	9.7 (8.9, 9.9)
CBT skills—actual use M (95% CI)	PSG	7.2 (5.5, 8.4)	8.2 (6.9, 9.0)	8.0 (6.4, 9.0)
	RL	9.3 (8.7, 9.7)	9.1 (8.2, 9.5)	9.1 (8.2, 9.6)
**Family Functioning #**				
General functioning	PSG	1.81 (1.50, 2.12)	1.91 (1.62, 2.21)	1.92 (1.60, 2.23)
	RL	1.97 (1.76, 2.19)	2.05 (1.83, 2.27)	2.00 (1.77, 2.24)
Communication	PSG	2.32 (2.04, 2.60)	2.13 (1.87, 2.39)	2.15 (1.87, 2.42)
	RL	2.24 (2.05, 2.43)	2.23 (2.03, 2.42)	2.19 (1.98, 2.40)
Problem-solving	PSG	2.26 (1.95, 2.58)	2.19 (1.88, 2.50)	2.31 (1.96, 2.66)
	RL	2.25 (2.03, 2.48)	2.16 (1.94, 2.39)	2.07 (1.81, 2.34)
**Support person outcomes**				
High perceived benefit ^a^–n (%)	PSG	1 (20.0)	2 (25.0)	0 (0)
	RL	5 (83.3)	3 (50.0)	1 (14.3)
Low burden ‡–n (%)	PSG	6 (100)	8 (100)	5 (100)
	RL	5 (83.3)	4 (100)	6 (100)
**Cancer Needs Questionnaire for Parents/Carers**				
Number of high/very high needs	PSG	0.6 (0.1, 4.8)	1.0 (0.2, 3.9)	0.7 (0.1, 4.0)
	RL	2.4 (0.6, 7.0)	0.2 (0.0, 1.1)	1.0 (0.2, 3.7)

* Results are presented as Mean (Confidence Interval) unless otherwise indicated. ^a^ Comprised “quite a bit” and “very much” benefit ratings.‡ Comprised “not at all” and “a little bit” burden ratings. ^1^ NB: Scores 0–9 are in the Normal range for DASS-21 Depression, and scores 0–7 in the normal range for DASS-21 Anxiety subscales. ^ CES scores—A higher total score indicates higher event centrality (range: 7 to 35). # McMaster Family Assessment Device: Clinical cut-off for the subscales were 2.0 for the general family functioning subscale, 2.2 for the problem solving subscale and 2.2 for the communication subscale [96,97]. Some data were missing for some participants.

## Data Availability

The data presented in this study are available upon reasonable request from the corresponding author, as is the full study protocol and the intervention materials. The data are not publicly available due to restrictions within the ethical approval.

## Supplementary Materials

The following are available online at https://www.mdpi.com/article/10.3390/cancers13102460/s1, S1: Example fidelity rating scales for independent raters; S2: Methodology for calculating cost of delivering Recapture Life online program; S3: Detail on composition of group members for each online group; S4: Positive (Figure a) and Negative (Figure b) Impact of Cancer outcomes from three-way analyses comparing Recapture Life, peer-support group and waitlist controls; S5: Positive and Negative Impact of Cancer outcomes plotted against participant age, by treatment group; S6: Coping strategy use and helpfulness by treatment arm; S7: Proportion of participants who reported actually using each CBT coping strategy; S8: Reported cancer-related needs at baseline on the Cancer Needs Questionnaire for Parents and Carers of AYAs; S9: Additional output related to AYAs’ absenteeism (a) and productivity (b); S10: Hospital admissions and Emergency Department presentations, by treatment arm; S11: Prevalence of medication use across the trial period in Recapture Life versus peer-support group.
