# Supplementary material for: Online, Group-Based Psychological Support for Adolescent and Young Adult Cancer Survivors: Results from the Recapture Life Randomized Trial
Source: Cancers (Basel). 2021 May 18;13(10):2460. doi: 10.3390/cancers13102460 (PMC8158368; doi:10.3390/cancers13102460)
Supplement: Supplementary file 1 [file cancers-13-02460-s001.zip › cancers-1186413-supplementary.pdf]

SUPPLEMENTARY MATERIALS

Supplementary Data S1. Example fidelity rating scales for independent raters

Recapture Life Fidelity Ratings – Module 1

Rater:_________ Group #_________

Quality of treatment ratings:

0	1	2	3	4	5	6	N/A
Unacceptable	Marginal	Low……Acceptable……..High					

Please indicate the quality of each component using the above scale. If not observed, please put N/A.

Treatment component	Quality
Introduce self, role and background and facilitate group introductions	
Flag what procedure is in case of technical difficulties	
Discuss importance of confidentiality and respect to others in group; discuss any 'group rules' group would like to establish; address contact with other group members (e.g., becoming 'Facebook friends')	
Discuss rationale for learning helpful coping skills to manage getting back to 'normal' after cancer; clarify level of disclosure expected in program	
Briefly discuss reason for having a workbook and home practice exercises	
Discuss range of 'normal' responses to cancer experience; normalise	
Introduce ABC model – explain basic model and cancer-related examples	
Introduce home practice for the week	
**Based on your review of this module, please indicate which arm you believe the module is from:**<br>**___ Peer support-group**<br>**___ Intervention (Recapture Life)**	

Supplementary Data S2. Methodology for calculating cost of delivering *Recapture Life* online program.

Delivery costs (Recapture Life personnel). The cost of delivering the *Recapture Life* and peer-support group online programs was calculated by analyzing a detailed log of all participant contacts relevant to the appropriate clinical-delivery of the Recapture Life program during the trial, including the number and length of all clinical contacts. We calculated costs differently according to whether they had been undertaken by trained study psychologists, or study research officers, and took into account some variability in the level of clinical psychologist and research officer appointed. All contacts made by either the study psychologists, or the research officers, that were focused on the delivery of the program were recorded, and the number and length of these were calculated. These included telephone calls/interviews by study psychologists for the purposes of assessment (e.g., intake assessment, or distress screen if participant returned a concerning Emotion Thermometers Tool between sessions), as well as the time spent by psychologists in delivering the online program. For research officers, this included telephone calls and text-message reminders to assist participants in accessing the Recapture Life online platform, reminding them of upcoming sessions, and inquiring as to their emotional state between sessions. Some of these tasks (e.g., online sessions, intake interviews) had actual times logged by the team; other tasks (e.g., an SMS reminder) were assigned an estimated time allocation by team consensus (e.g., five minutes for an SMS reminder/conversation; 30 minutes for a psychologist telephone call to screen for distress/risk). The frequency/number of these activities, and their length of time, were then calculated together with the cost of the individual delivering these. We calculated the cost of a research officer with an appropriate level of training within our University at a range of AU$39.26-40.55/hour. For the psychologist facilitators, the cost calculated was AU$50.86-53.92/hour, which provides a range of costs from a Year 1 Clinical

Psychologist employed within New South Wales Health, through to a clinical-academic psychologist within the University (A8 level), as well as the time of a Research Officer (UNSW Level 5.1 or 5.2). Salaries included 24% on-costs for the University positions and 18.7% on-costs for the New South Wales Health positions. Using these cost ranges as a base input, two modelling methods were used to get the most accurate estimation of costs.

Method 1: Modelling/estimation. In this approach, for the group tasks, we assumed that there would be a fixed amount of time needed for running a group session (no matter how many participants were there), plus some extra time that depended on the number of participants. We used a regression model to estimate both of these aspects for each intervention group, and then calculated the total (per-participant) time needed for 6 sessions, each with 3 participants in them. For non-group tasks (e.g. between-session emails and SMS text messages), we estimated a fixed time for a participant for their induction, in addition to some extra time that depended on the number of sessions they attended, and estimated these in each arm with a regression model. We used this to estimate the total time needed for an individual attending 6 sessions. This first method of estimation resulted in the following estimated costs:

Peer-support group: $367.06 - $400.02 per participant

Recapture Life: $485.58 – $531.45 per participant

Method 2: Simple average. We also estimated costs using a simpler method, for each group calculating the total time required for clinical intervention/contact components and divided this by the number of participants to get the per-participant times. For individual tasks, we considered participants who attended at least 4 sessions and scaled up their time as if they had attended all 6 (i.e., got an average per-session time and multiplied that by 6). We then added the individual times to the per-participant group times from each individual’s group, and then

took the average within each intervention arm. We used the same salary ranges as noted above to get the following range of costs in each arm:

Peer-support group: $380.86 - $413.87 per participant

Recapture Life: $491.24 - $537.42 per participant

Estimated travel costs saved for AYAs. We also estimated travel costs we were able to avert for our AYA participants. There is limited guidance around how best to estimate costs incurred to travel for cancer-related care (Molyneux, 2012). To achieve this, we calculated the estimated additional costs that would have been borne by participants if they had to travel to their hospital site to attend the weekly *Recapture Life* intervention sessions, rather than receiving the program online. We estimated the travel cost savings for participants, using the distance from the participant's home to the nearest capital city according to Google Maps. Firstly, we calculated the cost of fuel saved by using the method outlined by the Royal Automobile Club of Queensland. This method utilizes total trip distance, automobile fuel consumption rate, and price of fuel (https://www.racq.com.au/cars-and-driving/cars/owning-and-maintaining-a-car/fuel-saving-tips/estimating-the-cost-of-fuel-for-a-trip). This method uses the following equation to determine the cost of a trip: (Total trip distance in KM/100)*Fuel Consumption Rate*Price of fuel.

To estimate the total amount of savings for participations, we examined the RACQ reimbursement estimate per session and multiplied them by the total number of sessions participants would have had to attend (8 in total: intake, 6 weekly sessions, booster).

Supplementary Data S3. Detail on composition of group members for each online group

In our trial, we did not stratify groups by gender (i.e., all male and all female groups) by design, due to mixed/little evidence suggesting this would be beneficial. Additionally, while we originally intended to organize groups according to participant age, this was ultimately not possible due to the slower-than-expected pace of recruitment. The ultimate composition of our online group members is depicted in the table below.

Table. Composition of online Recapture Life and peer-support groups according to sex and age of group participants

	**RL1**<br>Girl 23<br>Girl 18	**RL2**<br>Girl 18<br>Girl 17	**RL3**<br>Girl 25<br>Boy 21<br>Girl 24	**RL4**<br>Boy 15<br>Girl 16<br>Girl 22	**RL5**<br>Girl 21<br>Boy 18<br>Girl 19<br>Boy 20	**RL6**<br>Girl 18<br>Girl 19<br>Boy 18<br>Boy 19	**RL7**<br>Boy 20<br>Boy 19<br>Boy 18
**Age difference (years)**	**5**	**1**	**4**	**7**	**3**	**1**	**2**
	**PSG1**<br>Boy 19<br>Boy 20<br>Girl 24	**PSG2**<br>Girl 25<br>Boy 20<br>Girl 19	**PSG3**<br>Boy 18<br>Boy 24<br>Girl 23<br>Girl 25	**PSG4**<br>Girl 22<br>Boy 25<br>Boy 18<br>Boy 23	**PSG5**<br>Girl 22<br>Boy 20<br>Boy 24<br>Girl 25	-	-
**Age difference (years)**	**5**	**6**	**7**	**7**	**5**		

As shown above, groups varied between 2-4 members each, with most groups (9/12, 75%) including a mix of genders. The group clusters varied in terms of the difference in ages the group members represented, with slightly over half (7/12, 58%) of the groups having members spanning 5 or more years difference in age, and the remainder (5/12 groups, 42%) having group ages that spanned between 1-4 years' difference.

Despite many of our groups involving AYAs whose ages spanned five or more years, this did not appear to impact the clinical and interpersonal processes involved in running the intervention. Our facilitators kept detailed session notes, and did not observe marked differences in the positive engagement in group processes according to whether or not group members had a greater age difference or not; rather, it seemed that often the AYA group members managed to find common ground through which to relate to their fellow group members even when there was a difference in gender or a few years' age.

We examined our group cohesion data plotted against the composition of our groups and were not able to observe any clear evidence of a signal that either the gender or age-related make-up of the groups was associated with the extent to which the group rated the group cohesion positively.

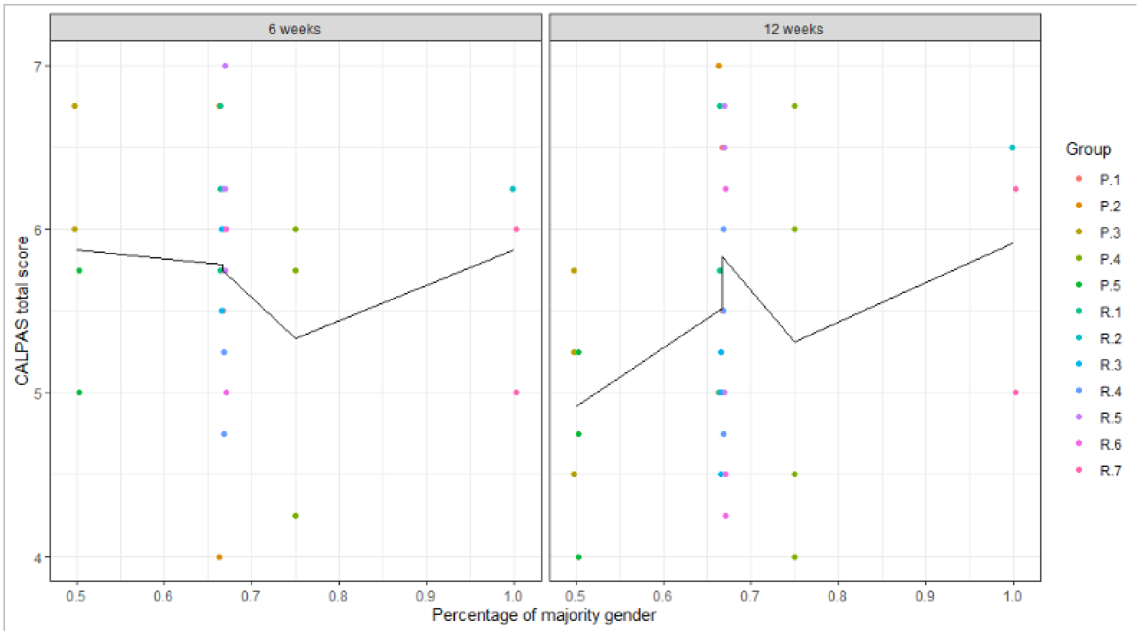

Figure: CALPAS scores plotted against the gender composition of the group (from an even 50:50 split through to groups comprised entirely of one gender)

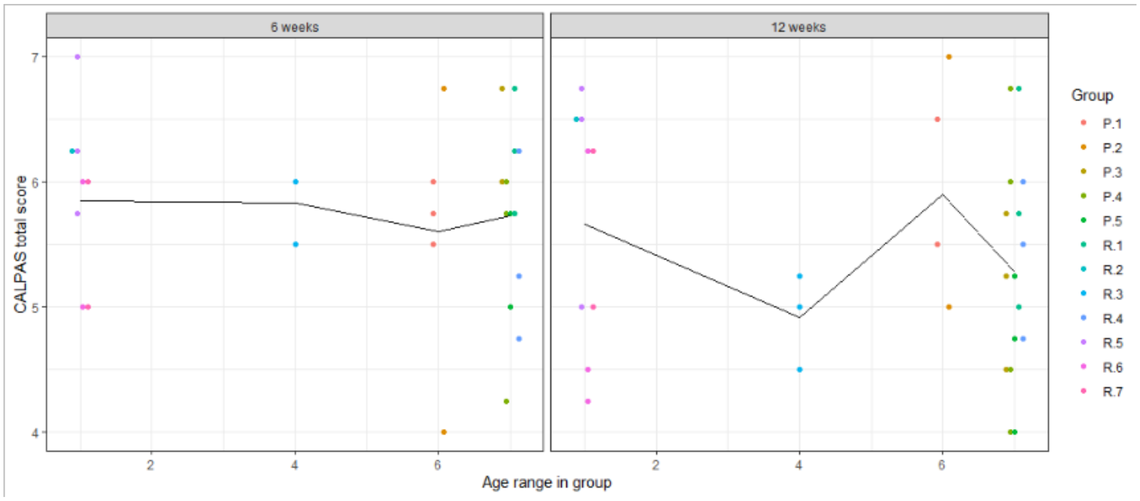

Figure: CALPAS scores plotted against each group's age range in years (from youngest-oldest member)

Supplementary Data S4. Positive (Fig. a) and Negative (Fig. b) Impact of Cancer outcomes from three-way analyses comparing *Recapture Life*, peer-support group and waitlist controls

a.

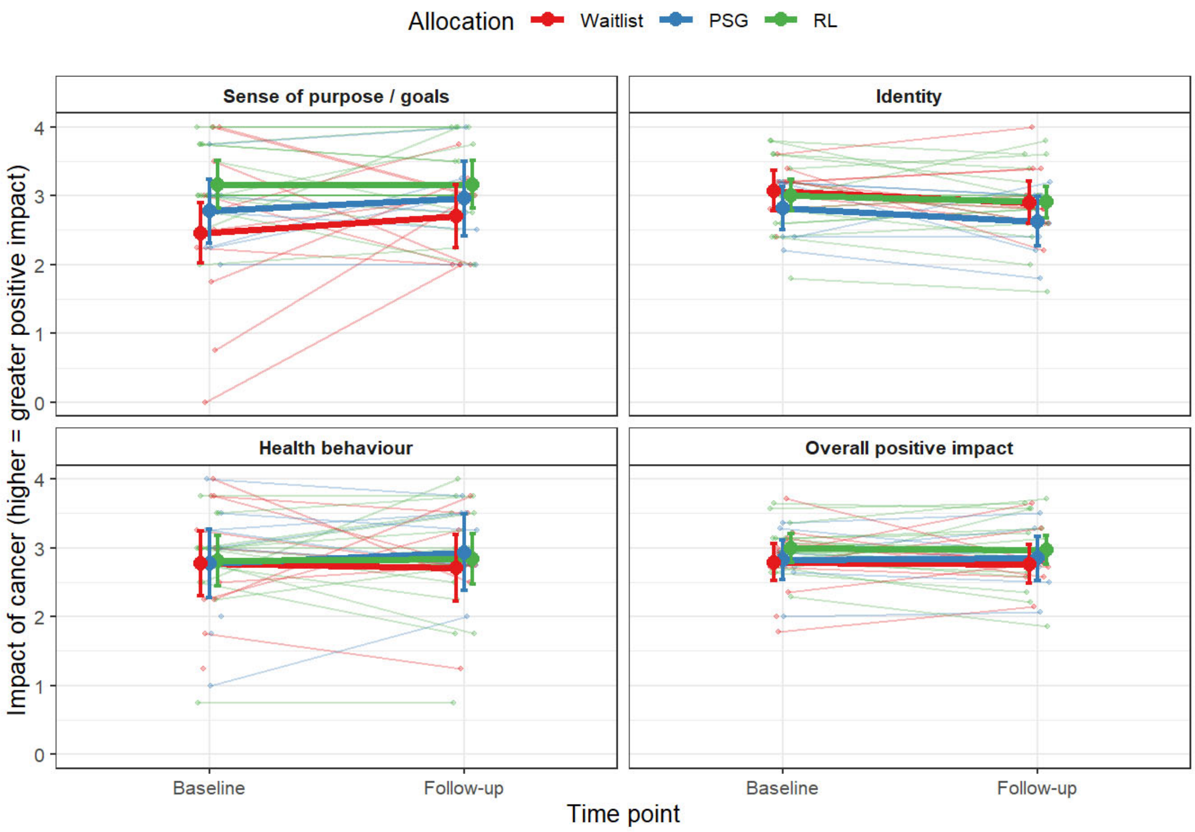

b.

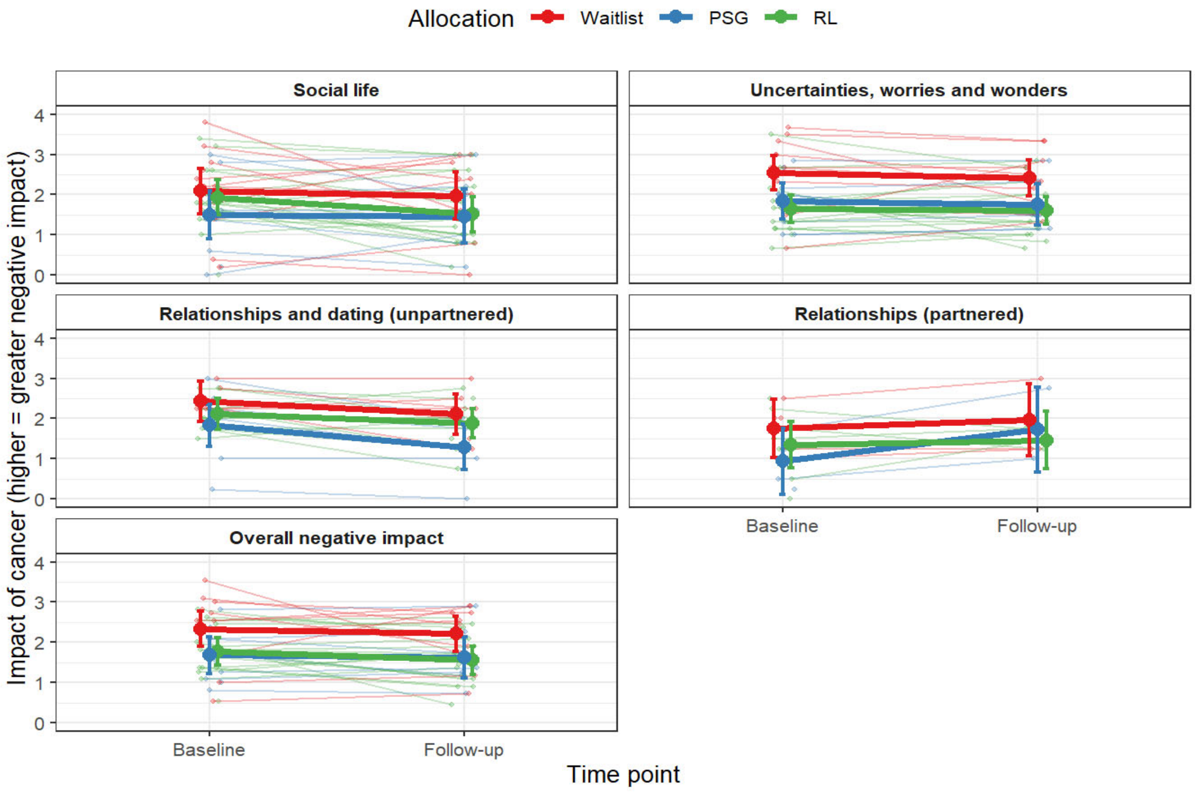

Allocation
Waitlist
PSG
RL
Social life
Uncertainties, worries and wonders
Relationships and dating (unpartnered)
Relationships (partnered)
Overall negative impact
Impact of cancer (higher = greater negative impact)
0
1
2
3
4
Baseline
Follow-up
Time point

Supplementary Data S5.Positive and Negative Impact of Cancer outcomes plotted against participant age, by treatment group

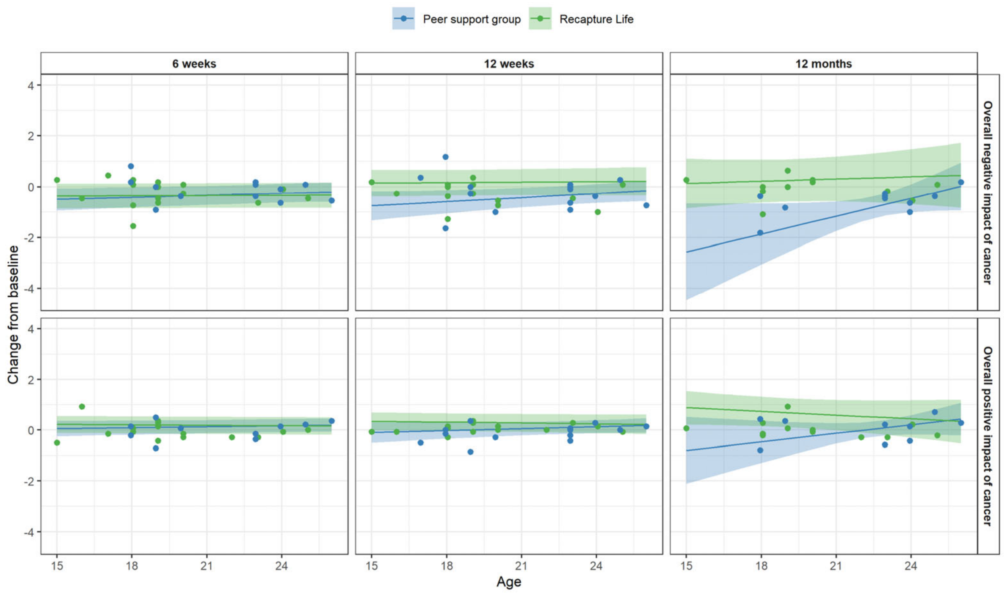

Supplementary Data S6. Coping strategy use and helpfulness by treatment arm

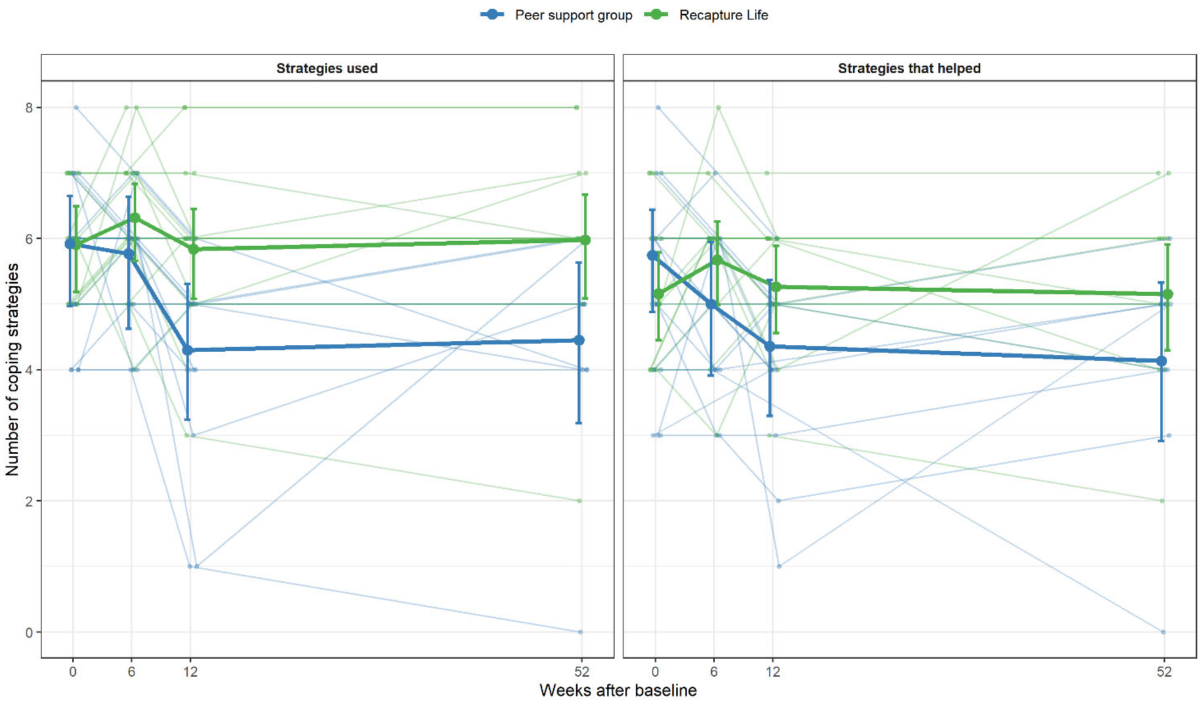

Supplementary Data S7.Proportion of participants who reported actually using each CBT coping strategy

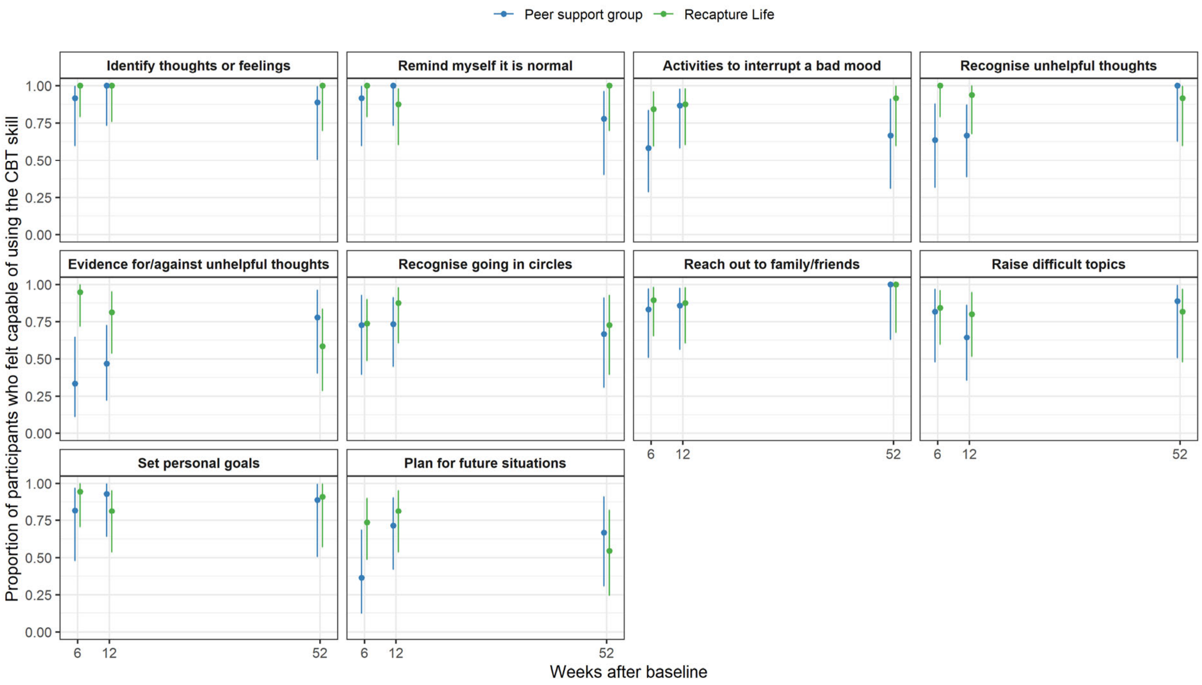

Supplementary Data S8. Reported cancer-related needs at baseline on the Cancer Needs Questionnaire for Parents and Carers of AYAs

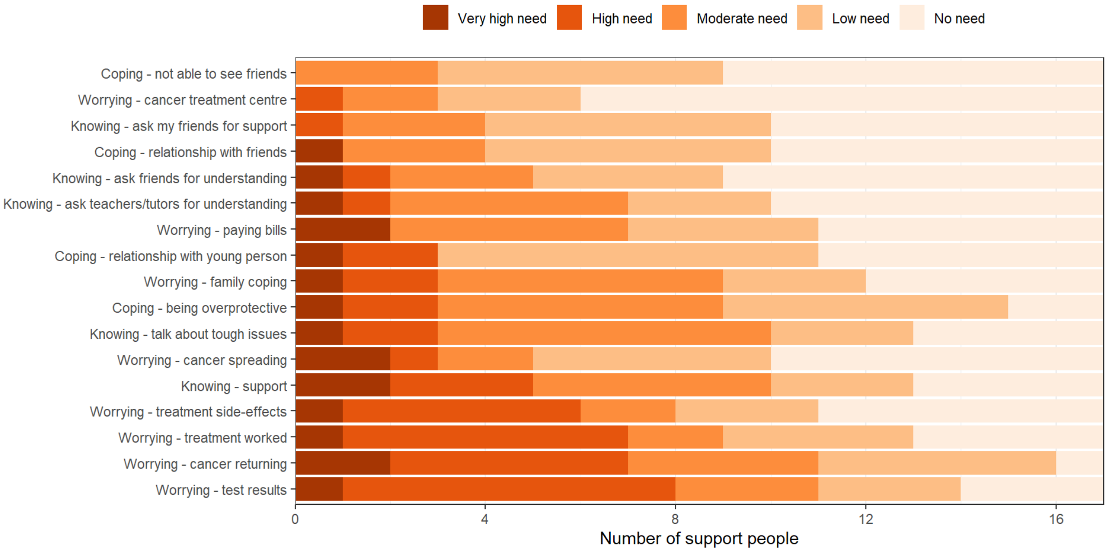

Supplementary Data S9. Additional output related to AYAs' absenteeism (a) and productivity (b)

a) Self-reported absenteeism from paid work and study, by treatment arm

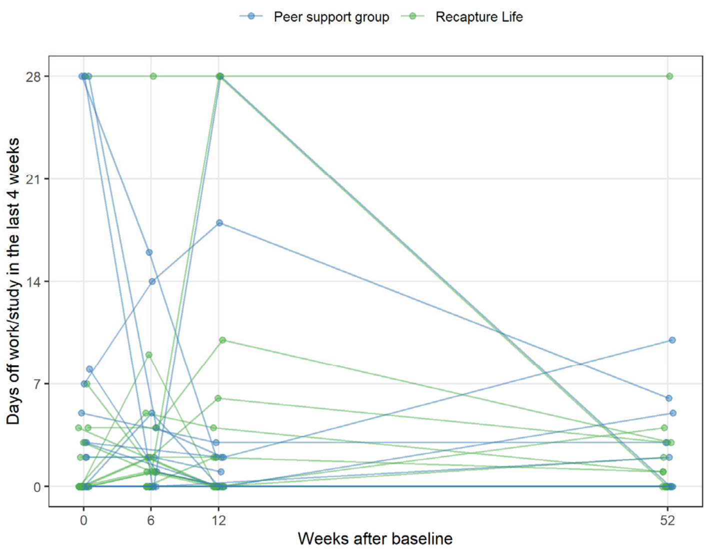

b) Variability in AYAs' engagement in different productive activities over time and according to treatment arm (*Recapture Life* vs. peer-support group)

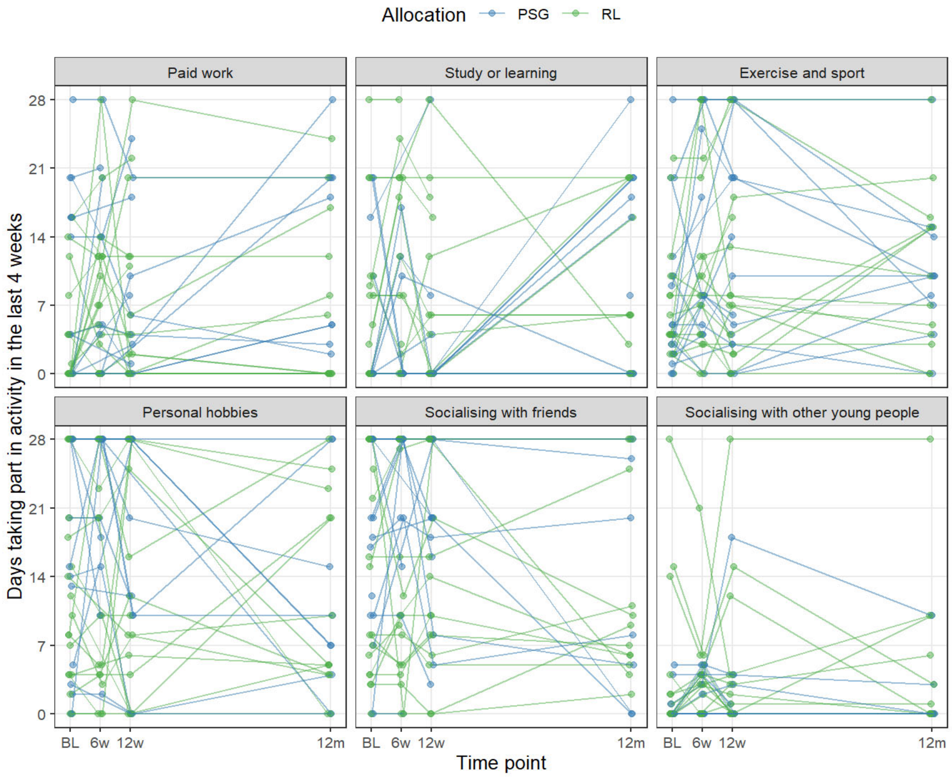

Note: PSG=Peer-support group, RL=Recapture Life, BL=Baseline, 6w=6-weeks, 12w=12-weeks, 12m=12 month follow up

Supplementary Data S10.Hospital admissions and Emergency Department presentations, by treatment arm

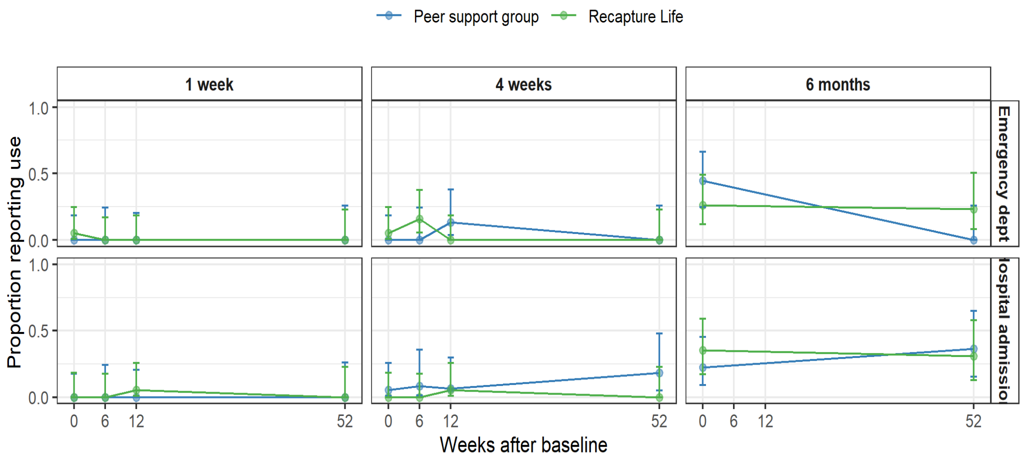

Supplementary Data S11. Prevalence of medication use across the trial period in Recapture Life versus peer-support group

	Peer-support group				Recapture Life			
	**Baseline (n=18)**	**6 weeks (n=11)**	**12 weeks (n=12)**	**12 months (n=10)**	**Baseline (n=19)**	**6 weeks (n=19)**	**12 weeks (n=16)**	**12 months (n=12)**
Any medication use	13 (72%)	6 (55%)	7 (58%)	5 (50%)	11 (58%)	11 (58%)	10 (62%)	8 (67%)
